# Bis(diethylamino)(pentafluorophenyl)phosphane – a Push–Pull Phosphane Available for Coordination

**DOI:** 10.1002/ejic.201100138

**Published:** 2011-04-27

**Authors:** Andreas Orthaber, Michael Fuchs, Ferdinand Belaj, Gerald N Rechberger, C Oliver Kappe, Rudolf Pietschnig

**Affiliations:** [a] Institute of Chemistry, University of GrazSchubertstr. 1, 8010 Graz, Austria, Fax: +43-316-380-9835; [b] Christian Doppler Laboratory for Microwave Chemistry and Institute of Chemistry, University of GrazHeinrichstrasse 28, 8010 Graz, Austria; [c] Institute of Molecular Biosciences, Department of Mass Spectrometry, University of GrazHeinrichstrasse 31, 8010 Graz, Austria

**Keywords:** Phosphorus, Fluorinated ligands, Homogeneous catalysis, Click chemistry

## Abstract

A facile large-scale synthesis of the “push–pull”-substituted ligand bis(diethylamino)(pentafluorophenyl)phosphane is reported. A selenophosphorane as well as complexes with CuI and PdCl_2_, can be formed almost quantitatively from suitable starting materials. The Pd^II^ complex shows a square-planar coordination with significant distortions of the Cl–Pd–Cl moiety in the solid state. In contrast, the phosphane ligand exhibits a large flexibility in the trigonal-planar coordination of the Cu salt, as proven by X-ray crystallography. C–C cross-coupling reactions and 1,3-dipolar cycloadditions have been tested for the Pd^II^ and Cu^I^ complexes, respectively. Whereas the reactivity of the Pd^II^ complex is good at low temperature, the Cu^I^ complex reveals remarkable reaction rates at temperatures up to 130 °C. Furthermore, the Cu^I^-catalyzed azide/alkyne 1,3-dipolar cycloaddition was successfully adapted for flow conditions.

## Introduction

Over the last decades palladium has achieved a prominent role in catalysis and organic synthesis. Different types of reaction protocols have been developed for the formation of C–C bonds by the use of palladium catalysts.^1^ In many cases optimized phosphane ligands play a crucial role in palladium catalysis, be it for electronic or steric reasons.^2^–^4^ Mono-, bis- and tris(amino)-substituted phosphanes represent electron-rich ligand systems,^5^ which have been investigated for instance in relation to hydrovinylation reactions.^5^ Especially (diethylamino)phosphanes have been widely studied with respect to their coordination behavior^6^ and their potential in different catalytic reactions.^7^,^8^

The opposite approach, that is the design of electron-deficient phosphane ligands also had a profound impact on catalyst design. Since the first preparation of (C_6_F_5_)_3_P in 1960^9^ and its structural characterization,^10^ a wealth of coordination compounds based on this ligand have been prepared.^11^ Thus, complexes with electron-withdrawing substituted phosphane ligands have been successfully tested in hydrocarbonylation, hydrocarboxylation, hydroformylation, and Suzuki coupling reactions yielding reasonable activities.^12^–^14^

It is generally accepted that in palladium-catalyzed cross-coupling reactions *cis*-phosphane-stabilized Pd^0^ species undergo oxidative addition with an aryl halide.^3^ In recent years, other more abundant metals with the same valence electron configuration (d^10^), for example Cu^I^, have also attracted significant attention in catalysis. In particular, Cu^I^-catalyzed Huisgen azide/alkyne 1,3-dipolar cycloaddition (CuAAC)^15^ has become a very popular variety of “click chemistry.”^15^–^19^ Nitrogen-based ligands have been used extensively to trigger these cycloaddition reactions.^20^–^22^ In the past few years also aminophosphane ligands have been used for this purpose, showing good stabilization of the Cu^I^ species involved.^23^,^24^

C_6_F_5_P(NEt_2_)_2_ was reported already in the 1960s^25^,^26^ and since then became a subject of occasional physicochemical studies,^27^–^29^ which contrasts with the rich chemistry of its nonfluorinated analogue, PhP(NEt_2_)_2_. Here we report a straightforward and practical alternative to the original synthetic procedure and further investigations into the coordination and catalytic chemistry of C_6_F_5_P(NEt_2_)_2_ in standard model reactions. Our findings are corroborated by X-ray crystallography, spectroscopic methods, and ab initio calculations.

## Results and Discussion

**Scheme 1:**
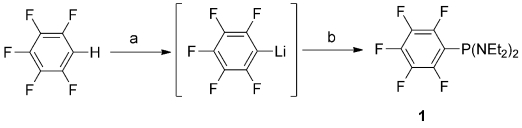
Synthesis of phosphane **1**. (a) *n*BuLi, –50 °C, Et_2_O; (b) –80 °C to room temp., ClP(NEt_2_)_2_.

Bis(diethylamino)(pentafluorophenyl)phosphane (**1**) combines electron-releasing amino groups with an electron-withdrawing fluoroaryl unit at the same phosphorus atom. Therefore, we were interested in the properties of this “push–pull”-substituted phosphane and in further exploring the reactivity of this class of compounds. Compound **1** was synthesized by the straightforward and cost-efficient reaction of LiC_6_F_5_ with ClP(NEt_2_)_2_ at low temperature yielding the pure product after distillation (Scheme 1). Spectroscopic data for **1** are in agreement with the available literature data and have been further amended.^28^

### Reactivity at the Phosphorus Atom

**Scheme 2:**
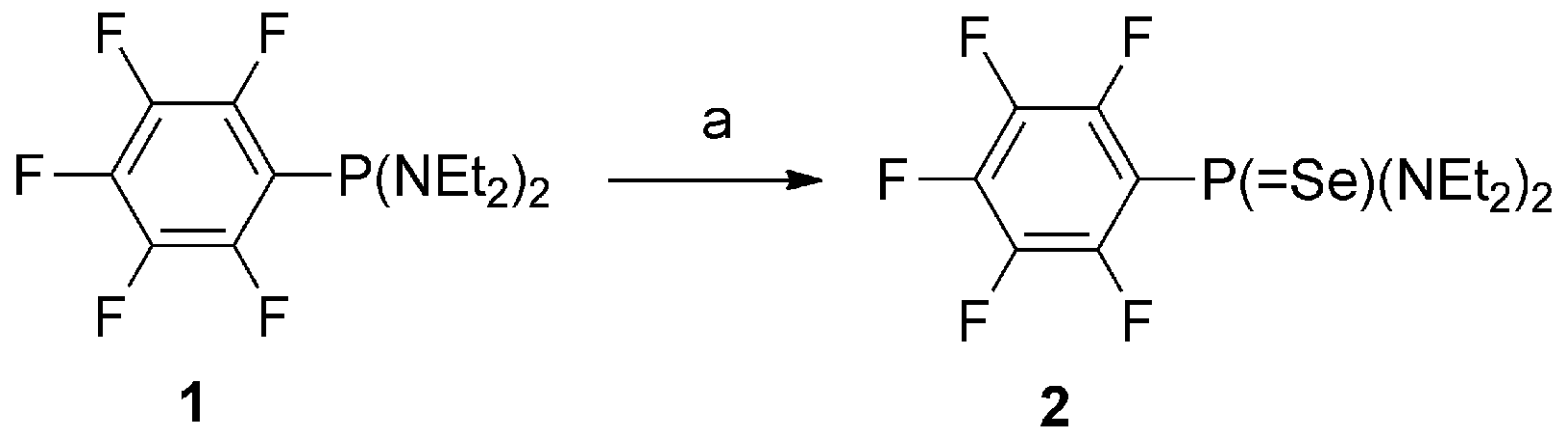
Formation of the selenophosphorane **2**. (a) CHCl_3_, room temp., 15 min, Se_gray_ (1.2 equiv).

**Figure 1:**
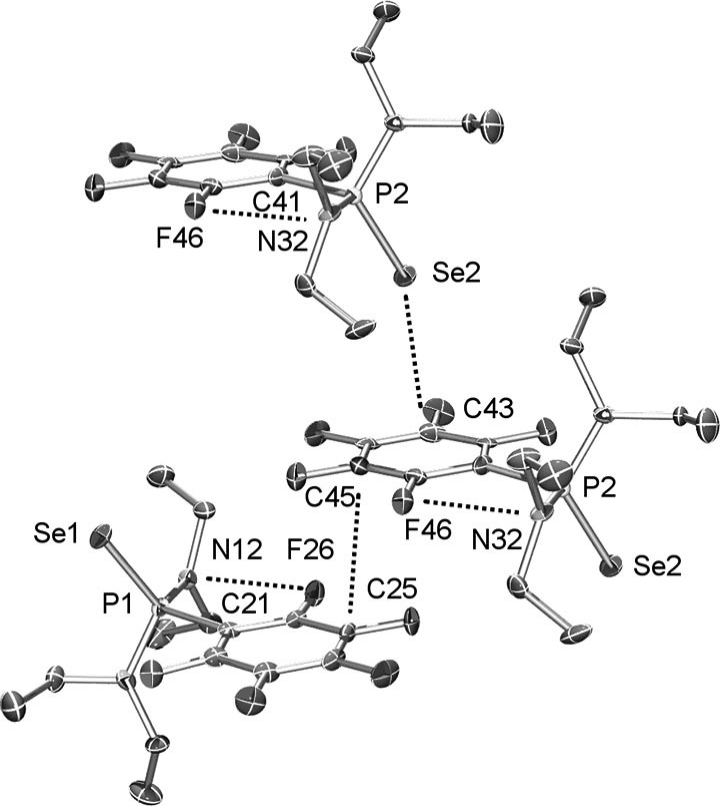
ORTEP drawing of the selenophosphorane **2** at a probability level of 50 %. Hydrogen atoms and the second unit are omitted for clarity. Intra- and intermolecular contacts are indicated by dashed lines. Selected distances [Å] and angles [°]: P1–Se1 2.102(2), P2–Se2 2.087(2), P1–C21 1.845(7), P2–C41 1.839(7), F26–N12 2.844(7), F46–N32 2.767(7), Se2–C43 3.346(7), C25–C45 3.329(10); C26–C21–P1–N12 –5.3(7), C46–C41–P2–N32 –10.7(7).

The closely related analog of **1** in which ethyl groups were replaced by isopropyl groups was reported in 2003.^6^ Interestingly, in case of the isopropyl derivative C_6_F_5_P(N*i*Pr_2_)_2_ neither formation of a selenophosphorane nor coordination to (carbonyl)Rh complexes could be achieved, which was attributed to steric and electronic factors.^6^ By contrast, the facile selenation of a further related phosphane, HC_6_F_4_P(NEt_2_)_2_, in which the amino groups are identical but the fluoroaryl substituent differs by one fluorine atom, was published very recently.^30^ In the light of this discrepancy, we wondered whether **1** would be unavailable for reactions at the lone pair, just like its isopropyl-substituted analog, or prone to oxidation and coordination like its partially fluorinated congener. In an initial attempt to explore the reactivity of the phosphorus lone pair in this compound we investigated the oxidation of **1** with gray selenium (Scheme 2). The envisaged selenophosphorane **2** is easily obtained at room temperature in quantitative yield. In the ^31^P NMR spectra a single resonance at *δ* = +49.4 ppm is observed showing the expected ^77^Se satellites (^1^*J*_PSe_ = 800 Hz), which is shifted to high field compared with **1**. In general, the ^1^*J*_PSe_ coupling constant in selenophosphoranes is a rough estimate for the overall electron-withdrawing property of the substituents at the phosphorus atom featuring larger values for more electron-withdrawing substituents.^31^ The coupling constant of 800 Hz in **2** is in the typical range for tris(alkylamino)selenophosphoranes,^32^ whereas the ^77^Se NMR signal of **2** resonates at lower field compared to (Me_2_N)_3_PSe, while the ^31^P NMR signal is observed at higher field {*δ*_Se_ = –136.9 ppm, *δ*_P_ = 49.4 ppm (**2**) vs. *δ*_Se_ = –366 ppm, *δ*_P_ = 81.8 ppm [(Me_2_N)_3_PSe]}. Selenophosphorane **2** shows similar spectroscopic data as its partially fluorinated analog HC_6_F_4_P(=Se)(NEt_2_)_2_.^30^ The facile reaction of **1** with elemental selenium is in marked contrast to the reactivity of the analogous *i*Pr_2_N-substituted phosphane reported by Dyer et al.^6^ Actually, our findings suggest that electronic deactivation by the fluoroaryl substituent is in fact not decisive. This means in turn that steric factors are most likely responsible for the quite drastic difference in reactivity on going from R = Et to R = *i*Pr in C_6_F_5_P(NR_2_)_2_. The selenophosphorane **2** could also be characterized by means of X-ray diffraction and by EI-MS showing the characteristic pattern for the selenium-containing species. Geometric parameters are similar as for HC_6_F_4_P(=Se)(NEt_2_)_2_.^30^ A remarkable feature of these compounds is the almost coplanar orientation of one P–N bond relative to the aromatic moiety, resulting in short F**···**N contacts [2.767(7) Å and 2.844(7) Å] for both molecules in the asymmetric unit. Furthermore, weak π–π and π–Se interactions can be concluded from the solid-state packing. A graphical representation of **2** is depicted in Figure 1. Our observation that the sterically less shielding diethylamino groups significantly enhance the reactivity in **1** should open the way to the coordination chemistry of this class of phosphane ligands.

### Coordination Behavior

**Scheme 3:**
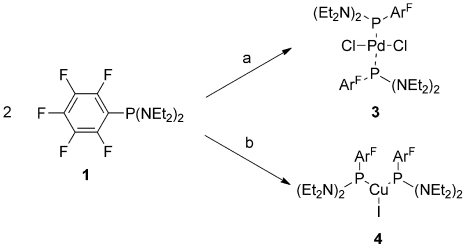
Synthesis of complexes **3** and **4** (Ar^F^ = C_6_F_5_). (a) Cl_2_Pd(CH_3_CN)_2_, CH_3_CN, room temp., 2 h; (b) CuI, CHCl_3_, room temp., 0.5 h.

In order to study the coordination behavior of phosphane **1** we investigated its reactivity towards late transition metals like Pd and Cu. The corresponding Pd complex (**3**) can be synthesized from a suitable precursor, that is Cl_2_Pd(MeCN)_2_, and ligand **1** in nearly quantitative yield within a short time (Scheme 3). Coordination of the free ligand **1** to the metal center is accompanied by a color change to intense orange. In the ^1^H NMR spectra of **3** the multiplet of the CH_2_ group splits into two distinctive multiplets. This diastereotopic effect for the protons of the methylene group upon coordination of the metal atom to phosphane **1** is comparable to that observed for benzylphosphanes.^33^

Coordination shifts (Δ*δ* = *δ*^31^P_complex_ – *δ*^31^P_free ligand_) have been studied by Trzeciak et al. showing a correlation between Δ*δ*, the phosphorus cone angle^34^ and the Pd–P bond strength.^35^ The coordination shift (Δ*δ*) in the ^31^P NMR spectra of this phosphane is quite small (Δ*δ* = –3.6 ppm; **1**: 82.0 ppm, **3**: 78.4 ppm). A single crystal structure analysis of Pd complex **3** confirms that in the solid state the ligand coordinates through the phosphorus atom to the metal center. Two independent molecules of complex **3** with very similar structural parameters are present in the unit cell. One is located near the center of inversion and shows disorder over two orientations, whereas the second molecule is not disordered. Consequently, the geometric parameters are discussed only for the molecule without disorder. The bis(diethylamino)(pentafluorophenyl)phosphane ligands in **3** are bonded to the Pd atoms in *trans* orientation. The pentafluorophenyl rings adopt orientations where an N atom of a diethylamino group is coplanar to the ring [N–P–C–C smaller than 1.8(2)°]. The most remarkable structural feature of **2** is the significant distortion from planarity around the central Pd atom. Whereas the P–Pd–P angle is almost linear [178.27(2)°], the Cl–Pd–Cl angle deviates strongly from linearity with 164.96(2)°.

**Figure 2:**
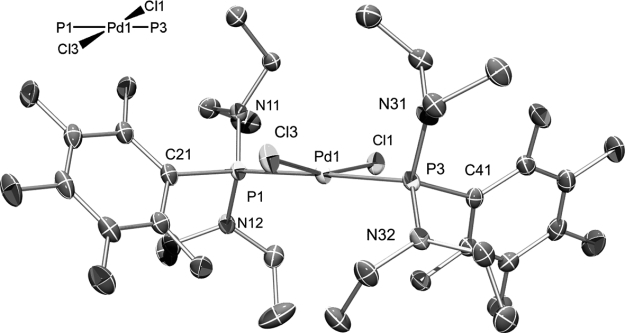
ORTEP drawing of Pd complex **3** at a probability level of 50 % and schematic inset illustrating the *trans* configuration. Hydrogen atoms and the second unit are omitted for clarity. Selected distances [Å] and angles [°]: Pd1–Cl3 2.3090(5), Pd1–Cl1 2.3199(5), Pd1–P3 2.3221(5), Pd1–P1 2.3554(5), P1–N11 1.646(2), P1–N12 1.662(2), P1–C21 1.855(2), P3–N32 1.651(2), P3–N31 1.655(2), P3–C41 1.861(2); Cl3–Pd1–Cl1 164.96(2), P3–Pd1–P1 178.27(2).

There are only a few examples in the literature with a bent linear arrangement of the phosphane ligands across the metal atom with a Cl–Pd–Cl angle significantly smaller than 180°. In these cases, such bending was imposed by the use of rigid bidentate ligands,^36^–^39^ which in one case is also supported by a chlorine atom bridging two different metal centers (Pd–Cl–Pt).^40^ In the crystal structure of **3** the least-squares (l.s.) plane through P1, Pd1, P3, and Cl1 encloses an angle with the Pd1–Cl3 bond of 14.86(3)°. This motif is best described as a distorted planar geometry, because the Cl–Pd–P angles are close to a right angle [87.87(2)° to 91.81(2)°]. The Pd–Cl distances [2.309(1) Å and 2.320(1) Å] are rather long for dichloridobis(phosphane)palladium complexes, with the exception of bridging chlorine atoms, that is Pd–Cl–Pd, which can have Pd–Cl distances up to 2.407(5) Å.^41^ The Pd–Cl bond observed in **3** is 0.11 Å longer than for example in PdCl_2_(PPh_3_)_2_ (= **M2**). The Pd–P contacts are 2.322(1) Å and 2.355(1) Å and thus in the upper range of Pd–P distances for this class of compounds.^42^ Compared with C_6_F_5_P(N*i*Pr_2_)_2_ [1.683(3) Å/1.669(4) Å]^6^ and *p*-C_6_F_4_[P(NEt_2_)_2_]_2_ [1.675(2) Å/1689(2) Å]^43^ the P–N bonds in **3** are shortened upon coordination to the metal center [1.646(2) Å–1.662(2) Å], whereas the P–C bond is essentially unaffected. In contrast, the crystal structure of tris(pentafluorophenyl)phosphane derivative[(C_6_F_5_)_3_P]_2_PdCl_2_ shows much shorter Pd–P and Pd–Cl distances [2.291(1) Å and 2.305(1) Å, respectively], and the palladium center shows an almost planar coordination geometry.^44^ The molecular structure of **3** is depicted in Figure 2, and further crystallographic data are given in the Supporting Information. In solution the spectrometric data are in good agreement with the solid-state structure. Different mass spectrometric methods [i.e., DI-EI (direct inlet electron ionization) and the mild DI-ESI] lead to fragmentation of the complex, and only the ligand and its fragments could be observed (i.e., Ar^F^H^+^, Ar^F+^–NEt_2_, C_6_F_5_PH^+^). In order to test the thermal stability of **3**, we performed ^31^P variable-temperature (VT) NMR experiments. Measurements of **3** from room temperature (room temp.) up to 70 *°*C in C_6_D_6_ solution showed that no decomposition occurs, and the complex is stable at these temperatures for at least 30 min. The integrity of the complex is further corroborated by the persistence of the two distinctive multiplets for the methylenic protons in the ^1^H NMR spectra at elevated temperatures (70 °C). Formation of a second isomer (i.e., the *cis* isomer) could not be detected within the limits of resolution and quantification in ^31^P NMR spectroscopy.

### Computational Study of the Coordination Geometry at Pd^II^

For a better understanding of the unusual geometry around the palladium center in complex **3** we investigated two model compounds by means of DFT calculations. In our model compounds **M1** {[C_6_F_5_(Me_2_N)_2_P]_2_PdCl_2_} the ethyl groups of **3** were replaced by methyl groups to avoid nonessential rotations around the CH_2_–CH_3_ bond. The DFT studies (B3LYP/6-31G*/LANL2DZ) were performed on two isomers of **M1**, namely *cis*- and *trans*-**M1**, which are compared to computational results for the structurally well-characterized (Ph_3_P)_2_PdCl_2_ (= **M2**). A detailed analysis of the molecular orbitals was carried out in order to understand the bonding situation of these complexes. A comparison of measured and calculated geometric parameters as well as a natural bond orbital (NBO) analysis should reveal the differences and commonalities of both complexes. The P–Pd–P and Cl–Pd–Cl angles in *trans*-**M1** are 178.9° and 168.8°, respectively, which differs only slightly from the experimental values obtained by X-ray analysis.^45^ Calculated bond lengths are generally slightly longer than the measured ones, nevertheless the experimental geometry is nicely reproduced by the calculations. Differences in the natural charges of **M1** with the well-known **M2** should reveal the effect of the electron-withdrawing group of the phosphane ligand. An NBO charge analysis on the C_6_F_5_-substituted phosphane ligand in **M1** results in a net charge of 1.46/1.48 a.u. on P and 0.39 a.u. on Pd. In complex **M2** a quite similar charge for Pd (0.40 a.u.) is found but a less positive P-center (1.09 a.u.), which is in line with the electron-withdrawing properties of the C_6_F_5_ group of ligand **1**. The natural charge of the chlorine atoms seems to be unaffected by the phosphane ligands (–0.55 and –0.56 for **M1** and **M2**, respectively). A comparison of the absolute gas phase energies of *cis*-/*trans*-**M1** shows that the *trans* isomer is just 15.3 kcal mol^–1^ lower in energy (Δ*G*^298^). Thus, formation of the *cis* isomer may occur easily with suitable reaction partners. Owing to the distorted geometry in *trans*-**M1** and **3**, the isomerization barrier is expected to be rather low. From our calculations the experimentally observed bending of the Cl–Pd–Cl unit can be reasonably reproduced suggesting that secondary interactions of the ligand with the Pd–Cl moiety are responsible for this distorted geometry. Furthermore, it can be excluded that the significant distortion from planarity results from crystal packing effects.

### Coordination to Cu^I^

To check how general the unusual geometric features in Pd complex **3** are, we set out to explore the coordination behavior of phosphane ligand **1** towards a d^10^ metal ion where little backbonding can be expected. We have chosen Cu^I^ to extend our study owing to its relevance in “click” chemistry. The Cu^I^ complex **4** of phosphane **1** was obtained by direct reaction of the ligand with Cu^I^ in a 2:1 ratio in chloroform or dichloromethane (Scheme 3). As previously observed for Pd complex **3**, the coordination shift of Cu complex **4** in the ^31^P NMR spectra is quite small (Δ*δ* = –8 ppm). Similar to complex **3**, the methylenic protons are diastereotopic. Slow evaporation of chloroform or dichloromethane under ambient conditions gave colorless hexagonal crystals of **4a**. Crystal structure analysis of the complex shows a trigonal-planar surrounding of the central copper atom with almost ideal parameters. The P–Cu–P angle [129.60(1)°] is distinctly larger than the I–Cu–P angles [113.62(1)° and 116.77(1)°]. The C_6_F_5_ groups are almost coplanar [7.41(7)°] suggesting that π-stacking may govern the relative orientation of the two phosphane ligands. Thus, the inter-aryl distance is as short as 3.074(2) Å, which is 0.10 Å shorter than the sum of their van der Waals (vdW) radii. The pentafluorophenyl rings adopt orientations in which the N atom of one diethylamino group is coplanar relative to the aryl ring [torsion angles N–P–C–C smaller than 4.86(15)°]. The N atoms are consequently below/above the least-squares plane spanned by I1, Cu1, P1, P2 [N11 –1.331(4) Å, N12 +1.396(4) Å, N21 –1.444(4) Å, N22 +1.298(4) Å]. The P–N bonds are slightly longer [1.651(1) Å–1.668(1) Å] than in complex **3**. The observed Cu–I distance [2.5476(2) Å] is in the normal range for this class of compounds. On the basis of the structure analysis, we conclude that the nitrogen atoms do not contribute to the stabilization of the copper atom.

Interestingly, compound **4** is completely air-stable and shows no formation of Cu^II^ species over months under ambient conditions. Its stability is, however, limited to nonacidic media owing to the lability of the P–N bonds toward acid-mediated bond cleavage. The EI mass spectra of complex **4** exclusively show the ligand and its fragments, whereas in the ESI spectra also some fragments (L^+^ – NEt_2_) and adducts of the complex (**4^+^** – I and **4^+^** + Cu) could be observed.

**Figure 3:**
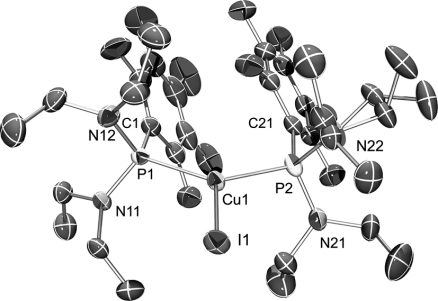
ORTEP drawing of complex **4a** at a probability level of 50 %. Hydrogen atoms are omitted for clarity. Selected distances [Å] and angles [°]: I1–Cu1 2.5476(2), Cu1–P1 2.2373(4), Cu1–P 22.2609(4); P1–Cu1–P2 129.60(1), P1–Cu1–I1 116.77(1), P2–Cu1–I1 113.62(1), I1–Cu1–P1–C1 –172.02(5), I1–Cu1–P2–C21 –177.59(5).

**Figure 4:**
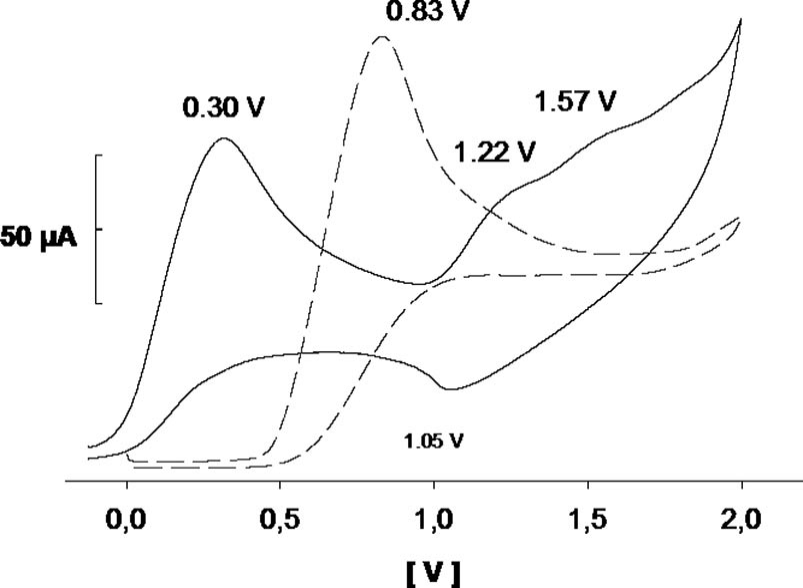
Cyclovoltammograms of **1** (dashed) and **4** (solid) in CH_3_CN (0.06 m Bu_4_N^+^BF_4_^–^) vs. Ag^0^/Ag^+^ (referenced externally to FcH/FcH^+^ at 0.96 mV) at a scan rate of 100 mV s^–1^.

In order to investigate the redox properties of this complex we performed cyclic voltammetry (CV) measurements and compared the results of **4** with those obtained for the free ligand (Figure 3). Ligand **1** exhibits a single oxidation wave at +0.83 V, whereas a single irreversible oxidation wave at +0.30 V dominates the cyclovoltammogram of complex **4**. The irreversible nature of the latter process again suggests a ligand oxidation, however now at lower potential owing to the vicinity of the metal cation. Further oxidation processes occur at potentials of +1.22 V and +1.57 V, which give a quasireversible single reduction wave at +1.05 V. The reversible nature of this process in combination with the potential range suggests that the couple Cu^I^/Cu^II^ may be involved probably bonded to a second ligand unit. In essence these data suggest that **1** and **4** should be stable against oxidation in air in the absence of water (Figure 4).

Since CuAAC (“click”) cycloaddition reactions are often carried out in donating/coordinating solvents, we wanted to explore the behavior of the complex towards solvents having oxygen (THF) and nitrogen (MeCN) donor sites. In solution no significant differences in the ^31^P NMR spectra can be observed. We were able to grow single crystals from both solvents. Although the copper atom still has a trigonal-planar coordination environment, the two phosphorus ligands show significantly different orientations. Whereas in **4a** both C_6_F_5_ groups of the ligands are in *trans* orientation to the iodide [Cu–I–P–C –172.02(5)° and –177.59(5)°] in compound **4b** (crystallization from MeCN) only one of the C_6_F_5_ rings adopts this *trans* orientation [Cu–I–P–C 175.00(4)°], and the other one is in a *cis* orientation [Cu–I–P–C –16.67(4)°]. The P–Cu–P angle is also slightly larger than in **4a**, but the sum of the angles around the Cu atom is for all complexes ca. 360° [**4a** 359.98°, **4b** 360.00°, **4c** 359.77°]. The Cu–I bond is also slightly longer in **4b**. The structure obtained by recrystallization from THF (**4c**) shows very similar structural parameters. Details of the structure solution for **4a**,**b**,**c** are given in the Supporting Information. Phosphane ligand **1** entails a high spatial flexibility in these copper complexes, which therefore should be predestined to adapt to a large variety of substrates in catalytic reactions.

### Catalytic Studies

**Scheme 4:**
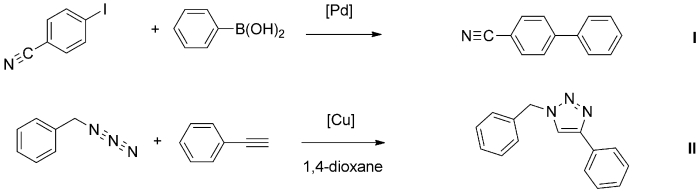
Reactions for catalytic studies. **I**: [Pd] 1.0 mol-% **3**, K_2_CO_3_, 1,4-dioxane, room temp. **II** [Cu] 2.5 mol-%, 1,4-dioxane, Δ*T*.

In order to test the catalytic scope of our push–pull phosphane complexes, we carried out two types of reactions. The catalytic performance of Pd complex **3** was studied with a Suzuki–Miyaura-type C–C cross-coupling reaction (Scheme 4) and compared to the activity of PdCl_2_(PPh_3_)_2_. Reactions were carried out under ambient conditions in 1,4-dioxane or toluene. The reaction conditions and achieved conversions are summarized in the Supporting Information. Neither solvents nor other reaction conditions (additional base, salts) were optimized for these catalytic studies. The stability of the complex against oxidation to P^V^ under ambient conditions is sufficient, and short exposure of **3** to air does not reduce its reactivity. At elevated temperatures (110 °C) under microwave heating palladium catalyst **3** shows an activity comparable to PdCl_2_(PPh_3_)_2_ and reactions without addition of ligand to PdCl_2_. Conducting these cross-couplings at ambient temperature shows superior conversion rates using **3** (i.e., time 48 h/74 % conv.) compared to the ones of the control experiments [PdCl_2_ (48 h/34 % conv.) and (PPh_3_)_2_PdCl_2_ (48 h/43 % conv.)]. The higher activity of **3** compared with PdCl_2_ and (PPh_3_)_2_PdCl_2_ is probably a result of the structural distortion discussed before associated with longer and consequently weaker and more reactive Pd–P and Pd–Cl bonds.

As outlined before, Cu^I^ species are often used as catalysts to perform CuAAC “click” reactions. Suitable catalysts are (amine-)stabilized Cu^I^ halides^46^ or can be generated in situ from Cu^II^ precursors and a reducing agent, that is CuSO_4_ and sodium ascorbate,^47^ or by synproportionation of Cu^0^ and Cu^II^. Elemental copper exhibits much better performance upon pretreatment with H_2_O_2_, suggesting Cu_2_O as the active species.^48^ Thus, we wanted to explore a homogeneous 1,3-dipolar cycloaddition reaction with **4** as catalyst. Initial studies in a microwave reactor on a model reaction (Scheme 4, II) demonstrated that **4** nicely triggers the CuAAC process (Table 1), whereas conversions without ligand (5 mol-% CuI) are below the detection limits (Entries 1, 3, 5, 7). The catalytically active species may be formed in situ from phosphane **1** with CuI showing excellent conversions (Entries 2, 4, 6, 8). Full conversion is reached at 60 °C after 90 min (Entry 6). Catalyst **4** or in situ generated catalyst give comparable results for conventional heating at 65 °C (Entries 9, 10). Full conversion at higher temperatures is already reached after 15 min (entry 11). Preliminary attempts to detect adducts of the copper catalyst **4** and either the alkyne or the azide by means of NMR spectroscopy have been unsuccessful. In the absence of azide, Glaser-type coupling reactions have been observed at ambient conditions with formation of the corresponding bis(alkyne), which could be proven by X-ray analysis. This suggests a competitive reactivity between Glaser-type and “click” reactions, which seems much faster for the latter, as evidenced by the complete lack of homocoupling products in the presence of azide.

**Table 1:** CuAAC of benzyl azide and phenyl acetylene. Reaction conditions: benzyl azide (0.5 mmol), phenyl acetylene (1.1 mmol); o.b.: conventional oil-bath heating; MW: microwave heating

Entry		Additive	Temp. [°C]	Time [min]	Conv. [%][a]	Yield [%][b]
1[c]	MW	–	60	5	<1	<1
2[c]	MW	**1**	60	5	21	16
3[c]	MW	–	60	45	<1	<1
4[c]	MW	**1**	60	45	70	76[f]
5[c]	MW	–	60	90	<1	<1
6[c]	MW	**1**	60	90	98	>99[f]
7[c]	MW	–	60	120	<1	<1
8[c]	MW	**1**	60	120	>99	>99
9[d]	o.b.	**4**	65	90	–	98
10[e]	o.b.	**1**	65	90	–	97
11[e]	o.b.	**4**	100	15	–	98

[a] Conversions determined by a calibrated HPLC-UV analysis.

[b] Isolated yields with NMR purity >95 %.

[c] Carried out in dioxane (2 mL), MW, 60 °C; compound **1** (5 mol-%) and CuI (5.0 mol-%) were added.

[d] Carried out in dioxane (2 mL), o.b., 2.5 mol-% **4** was added.

[e] Carried out in dioxane (2 mL), o.b., compound **1** (5 mol-%) and CuI (5.0 mol-%) were added.

[f] Contains impurities of **1**.

Owing to the stability of the catalyst at temperatures up to 100 °C, we envisaged to adapt the CuAAC process (Scheme 4, II) for continuous-flow conditions. A particularly attractive feature of the continuous-flow technology is the ease of scaling reaction conditions – without the need for reoptimization – through the operation of multiple systems in parallel (numbering-up, scaling-out), thereby achieving production-scale capabilities.^49^ Owing to the good performance of Cu complex **4** for this model reaction at higher temperatures, we explored the possibility to adapt this catalyst system for continuous-flow conditions. Previous studies on CuAAC chemistry performed in flow exclusively employed immobilized Cu^I^ species.^48^,^50^ The reaction was carried out by using a high-temperature capillary reactor (X-Cube Flash, ThalesNano).^51^ Best results were obtained at 130 °C and 4 mL min^–1^ flow rate, in which case a conversion of 80 % and a similar isolated yield was obtained (75 %). Hence, Cu^I^ complex **4** seems to be an excellent catalyst for CuAAC reactions over a wide range of temperatures suitable for continuous-flow applications in high-throughput or combinatorial chemistry. As an additional advantage, simple removal of the catalyst can be envisaged by acidic cleavage of the amino groups and subsequent aqueous extraction,^48^ or by fluorous extractions.^52^

The good activity of the complex is mainly attributed to the excellent stabilization of the Cu^I^ species. This is also underlined by the fact that solid complex **4** can be stored at ambient conditions in air over months, and neither decomposition nor disproportionation reactions are observed.

## Conclusions

We have demonstrated the cost-efficient synthesis of the pentafluorophenyl-substituted aminophosphane ligand **1**, which is available for reactions at the phosphorus lone pair. Besides the selenation also the coordination of **1** towards Pd^II^ and Cu^I^ centers has been accomplished, which is in marked contrast to its isopropyl analogue as described in the literature. On the basis of the distorted geometry of the Pd^II^ complex, which was confirmed by X-ray and theoretical studies, we concluded good reactivity in C–C coupling reactions. This was confirmed for Suzuki–Miyaura-type cross-coupling reactions at ambient conditions. Therefore, this catalyst is a promising candidate for C–C coupling reactions of temperature-sensitive compounds. The analogous Cu^I^ complex shows a trigonal-planar conformation for the central atom, whereas orientation of the ligands strongly depends on the coordination properties of the solvent. The catalytically active species was either added directly or prepared in situ showing equally good performance in CuAAC reactions, conducted under batch or continuous-flow conditions. Further studies must be carried out to investigate the scope and the limitations on substrates and reaction conditions of this push–pull phosphane ligand system.

## Experimental Section

**General:** NMR spectroscopic data were collected either with a Varian INOVA 400, or a Bruker Avance 300 operating at a proton frequency of 400 or 300 MHz, respectively; ^1^H and ^13^C NMR spectra were referenced internally to residual solvent signals; ^31^P and ^19^F NMR spectra were referenced externally to H_3_PO_4_ (85 %) and C_6_H_5_CF_3_ (*δ* = –63.7 ppm), respectively. Compound **1** was prepared according to a modified literature procedure.^6^,^25^ All reactions were carried out under an inert gas by using modified Schlenk techniques or a glove box. Solvents were dried with Al_2_O_3_ columns by the PureSolv solvent purification system. All chemicals were purchased from Aldrich or ABCR (C_6_F_5_H) and used without further purification. Owing to the evolution of HF, the determination of conventional combustion analyses was not possible with the equipment available to us. Therefore, the purity of products was determined with NMR, HPLC, or GC, and relevant spectra are depicted in the Supporting Information.

**Electrochemical Studies:** CV spectra were recorded with a Gamry Reference 600 device in CH_3_CN solution (Bu_4_NBF_4_ 0.06 m) of the respective substance under argon. A standard three-electrode system [Ag^+^/Ag reference electrode, Pt auxiliary wire, Pt working electrode (diameter 3 mm)].

**MS Measurements:** DI-EI measurements were performed with an Agilent 5975C quadrupole mass spectrometer equipped with a direct injection unit. HRMS measurements: Samples were dissolved in CH_3_CN and directly infused into the ESI ion source of a Synapt HDMS Q-TOF mass spectrometer (Waters, Manchester, UK). Samples were analyzed in positive-ion mode with a capillary voltage of 2.50 kV and a source temperature of 100 °C. The [MH]^+^ peak at *m*/*z* = 556.2771 of Leu-Enk (Sigma, 100 pg μL^–1^ in H_2_O/ACN, 1:1, v/v, 0.1 % HCOOH) was used as the reference signal. A table containing selected HRMS data is given in the Supporting Information.

**Preparation of Bis(diethylamino)(pentafluorophenyl)phosphane (1):** ***CAUTION! The lithio intermediate in the preparation of compound 1 is highly reactive and prone to LiF elimination at temperatures above –50 °C under the formation of very reactive aryne species!*** To a cooled (–50 °C) solution of C_6_F_5_H (7.58 g, 45.0 mmol) in THF (120 mL) was added *n*BuLi (28.3 mL, 45.3 mmol) over a period of 15 min, and stirring was continued at this temperature for 1 h. The suspension was cooled to –80 °C, and ClP(NEt_2_)_2_ (9.50 g, 45.1 mmol) was added slowly. The mixture was allowed to reach room temp. and stirred overnight. After removal of the solvent, the residue was extracted with pentane yielding crude **1**. Distillation under reduced pressure (89 °C, 0.026 mbar) yielded 80 % pure product (12.30 g, 35.9 mmol). ^1^H NMR (C_6_D_6_, 300 MHz, 298 K): *δ* = 0.97 (t, ^3^*J*_HH_ = 7.1 Hz, 12 H), 2.93 (tt, ^3^*J*_HH_ = 7.1 Hz, ^4^*J*_HF_ = 10.4 Hz) ppm. ^13^C NMR (C_6_D_6_, 75.5 MHz, 298 K): *δ* = 14.94 (d, ^3^*J*_PC_ = 3.5 Hz), 44.69 (d, ^2^*J*_PC_ = 19.2 Hz), 117.11 (dt, ^1^*J*_PC_ = 52.6 Hz, ^3^*J*_CF_ = 23.4 Hz; pseudo q, ^4^*J*_FC_ = ^5^*J*_CF_ = 3.7 Hz), 137.63 (dm, ^1^*J*_CF_ = 232.8 Hz), 140.95 (dm, ^1^*J*_CF_ = 229.5 Hz), 146.21 (dm, ^1^*J*_CF_ = 243.8 Hz) ppm. ^19^F NMR (C_6_D_6_, 282.4 MHz, 298 K): *δ* = –136.96, –156.48, –163.02 ppm. ^31^P NMR (C_6_D_6_, 121.5 MHz, 298 K): *δ* = 82.0 ppm.

**Preparation of Bis(diethylamino)(pentafluorophenyl)(seleno)phosphorane (2):** To a solution of **1** (920 mg, 2.7 mmol) in chloroform (2 mL) a slight excess of gray selenium (255 mg, 3.2 mmol, 1.2 equiv.) was added. The suspension was stirred at ambient temperature for 15 min. After filtration and removal of all volatiles, pure product was obtained in almost quantitative yield as a colorless solid (92 %, 1.04 g, 2.5 mmol). M.p. 56–58 °C (CHCl_3_). ^1^H NMR (CDCl_3_, 400 MHz, 298 K): *δ* = 1.18 (t, ^3^*J*_HH_ = 7.1 Hz, 12 H), 3.28 (m, 8 H). ^13^C NMR (CDCl_3_, 75.5 MHz, 298 K): *δ* = 14.08 (d, ^3^*J*_PC_ = 2.7 Hz), 44.69 (d, ^2^*J*_PC_ = 5.1 Hz), 113.32 (dt, ^1^*J*_PC_ = 91.6 Hz, ^3^*J*_CF_ = 18.4 Hz), 137.84 (dm, ^1^*J*_CF_ = 252.7 Hz), 142.85 (dm, ^1^*J*_CF_ = 254.1 Hz), 146.09 (dm, ^1^*J*_CF_ = 251.3 Hz) ppm. ^19^F NMR (C_6_D_6_, 282.4 MHz, 298 K): *δ* = –133.72 (m, *o*-F), –150.52 (m, *p*-F), –160.65 (m, *m*-F) ppm. ^31^P NMR (CDCl_3_, 145.1 MHz, 298 K): *δ* = 48.4 (s, ^1^*J*_PSe_ = 800 Hz) ppm. ^77^Se NMR (C_6_D_6_, 57.2 MHz, 298 K): *δ* = –136.9 (d, ^1^*J*_PSe_ = 800 Hz) ppm.

**Preparation of *trans*-Dichloridobis[bis(diethylamino)(pentafluorophenyl)phosphane]palladium(II), *trans*-Pd[P(C_6_F_5_)(NEt_2_)_2_]_2_Cl_2_ (3):** A mixture of PdCl_2_ (29 mg, 0.16 mmol) and acetonitrile (MeCN, 5 mL) was refluxed, until the brown residue was completely dissolved and the (MeCN)_2_PdCl_2_ complex formed. The solution was allowed to reach room temperature and subsequently added to a solution of **1** (112 mg, 0.33 mmol) in acetonitrile (3 mL); the mixture was stirred at room temperature for 2 h. The solvent was removed and the residue extracted with dichloromethane to yield **3** as an oily material. Recrystallization from cold MeCN (–28 °C) yielded 95 % (134 mg, 0.16 mmol) spectroscopically pure **3**. ^1^H NMR (C_6_D_6_, 300 MHz, 298 K): *δ* = 1.04 (t, ^3^*J*_HH_ = 7.0 Hz, 24 H), 3.19 (m, 8 H), 3.30 (m, 8 H) ppm. ^13^C NMR (C_6_D_6_, 75.5 MHz, 298 K): *δ* = 14.11 (br. s, CH_3_), 43.23 (br. s, CH_2_), 111.13 (tm, ^3^*J*_CF_ = 21.1 Hz), 137.54 (dm, ^1^*J*_CF_ = 253.8 Hz), 142.22 (dm, ^1^*J*_CF_ = 242.5 Hz) 146.80 (dm, ^1^*J*_CF_ = 249.8 Hz) ppm. ^19^F NMR (C_6_D_6_, 282.4 MHz, 298 K): *δ* = –132.28 (br. d, *J* = 20.5 Hz), –151.46 (br. tt, *J* = 21.9, 4.0 Hz), –162.43 (br. m) ppm. ^31^P NMR (C_6_D_6_, 121.5 MHz, 298 K): *δ* = 78.4 (br. s) ppm.

**Preparation of Bis[bis(diethylamino)(perfluorophenyl)phosphane]iodidocopper(I), Cu[P(C_6_F_5_)(NEt_2_)_2_]_2_I (4):** To **1** (349 mg, 0.70 mmol) in CDCl_3_ (700 μL) was added CuI (68 mg, 0.36 mmol) and the mixture stirred for 30 min. After filtration and removal of the solvent, pure product was obtained in quantitative yield (99 %, 302 mg). ^1^H NMR (CDCl_3_, 300 MHz, 298 K): *δ* = 1.04 (t, ^3^*J*_HH_ = 7.0 Hz, 24 H, CH_3_), 3.10 (m, 8 H, CH_2_), 3.20 (m, 8 H, CH_2_) ppm. ^13^C (100 MHz, CDCl_3_, 298 K): *δ* = 14.23 (s,CH_3_), 43.30 (br. s, CH_2_), 113.88 (br. s), 137.32 (dm, ^1^*J*_CF_ = 194.1 Hz, *m*-Aryl), 141.14 (dm,^1^*J*_CF_ = 255.3 Hz, *p*-Aryl), 145.62 (dm, ^1^*J*_CF_ = 246.0 Hz, *o*-Aryl) ppm. ^19^F NMR (CDCl_3_, 282.4 MHz, 298 K): *δ* = –135.01 (m), –152.70 (m), –161.28 (m) ppm. ^31^P NMR (C_6_D_6_, 161.9, 298 K): *δ* = 74.0 ppm.

**Catalytic Studies**

**Example Procedure for Suzuki–Miyaura-Type Cross Coupling:** Scheme 4. 4-Iodobenzonitrile (229 mg, 1 mmol), phenylboronic acid (145 mg, 1.2 mmol), potassium carbonate (207 mg, 1.5 mmol), and compound **2** (8.5 mg, 0.01 mmol) were weighed into a 10-mL Biotage microwave vial. Dry toluene (2.0 mL) was added, and the vial was capped with a Teflon-coated rubber septum and crimped. The reaction mixture was stirred for the time indicated in the Supporting Information, and samples (10 μL) were taken by syringe and cannula, diluted in MeOH (1 mL), filtered through a syringe filter, and subjected to GC-FID analysis. Further details (conditions and yields) are provided in the Supporting Information.

**Isolation of 4-Cyanobiphenyl:** The reaction mixture was stirred for 168 h achieving a GC-FID conversion of 90 %. The reaction mixture was filtered through a syringe filter, the solvent was removed under reduced pressure, the residue was dissolved in dichloromethane (1.5 mL) and adsorbed on a silica samplet of the Biotage SP1 system. After drying the samplet at 50 °C for 2 h, automated chromatography using a petroleum ether/EtOAc gradient (0–40 %) gave 4-cyanobiphenyl as a colorless solid (149 mg, 83 %) with the following physical properties identical to a reference sample: M.p. 84 °C (ref.^54^ 84–85 °C). ^1^H NMR (300 MHz, CDCl_3_, 298 K): *δ* = 7.76–7.68 (m, 4 H), 7.62–7.60 (m, 2 H), 7.53–7.44 (m, 3 H) ppm. ^13^C NMR (75 MHz, CDCl_3_, 298 K): *δ* = 145.7, 139.2, 132.6, 129.1, 128.7, 127.8, 127.3, 119.0, 110.9 ppm.

**General Procedure for the Copper-Catalyzed Cycloaddition** (**MW Conditions):** Scheme 4. Compound **3** (11 mg, 0.013 mmol) was weighed into a 10-mL Biotage microwave vial. 1,4-Dioxane (2.0 mL), benzyl azide (64 μL, 68 mg, 0.5 mmol), and phenyl acetylene (60 μL, 56 mg, 0.55 mmol) were added, and the vial was capped with a Teflon-coated rubber septum and crimped. The vial was placed into a microwave reactor (Biotage Initiator 2.5), and the reaction mixture was subjected to microwave irradiation by using the following settings: *T* = 60 °C, *t* = 1.5 h, prestirring = 10 s, sample absorption = normal. After the irradiation was finished, a sample (50 μL) was collected and subjected to HPLC analysis to determine the conversion. The remaining solution was concentrated under reduced pressure to yield 1-benzyl-4-phenyl-1,2,3-triazole (**9**) with slight ligand contaminations [122 mg, 2.5 % ligand contamination (NMR impurity), 98 %] with the following physical properties: M.p. 132 °C (ref.^55^ 132–133 °C). ^1^H NMR (300 MHz, DMSO, 298 K): *δ* = 8.67 (s, 1 H), 7.86 (m, 2 H), 7.52 (m, 8 H), 5.65 (s, 2 H) ppm. ^13^C NMR (75 MHz, DMSO, 298 K): *δ* = 147.1, 136.5, 131.1, 129.4, 129.3, 128.6, 128.4, 125.6, 122.0, 53.5 ppm.

**General Procedure for the Copper-Catalyzed Cycloaddition** (**Batch Conditions, Conventional Heating):** Scheme 4. Compound **3** (11 mg, 0.013 mmol) was weighed into a 10-mL Biotage vial. 1,4-Dioxane (2.0 mL), benzyl azide (64 μL, 68 mg, 0.5 mmol), and phenyl acetylene (60 μL, 56 mg, 0.55 mmol) were added, and the vial was capped with a Teflon-coated rubber septum and crimped. The solution was stirred for the indicated time at the respective temperature (see Table 1). The solution was concentrated under reduced pressure to yield 1-benzyl-4-phenyl-1,2,3-triazole with slight ligand contaminations (122 mg, 2.5 % ligand contamination purity was checked by NMR, 97.5 % pure product according to NMR analysis with identical physical properties as reported above).

**General Procedure for the Copper-Catalyzed Cycloaddition Under Flow Conditions:** An X-Cube flash flow reactor, equipped with a 16 mL coil, was adjusted to the following settings: *T* = 130 °C, 4.0 mL min^–1^ flow, *p* = 80 bar. Compound **3** (11 mg, 0.013 mmol), benzyl azide (64 μL, 68 mg, 0.5 mmol), phenyl acetylene (60 μL, 56 mg, 0.55 mmol), and 1,4-dioxane (2.0 mL) were combined in a 5-mL vial. As the reactor reached the reaction conditions, the mixture was introduced into the flow system. After the introduction, the pumps were switched back to the solvent reservoir, and 64 mL of solvent was collected in a 100-mL round-bottomed flask. The experiment was stopped, the solvent was removed under reduced pressure, and a sample of the remaining solid was subjected to NMR analysis to determine the conversion (80 %). The residual reaction mixture was taken up in acetone (2 mL), the product was precipitated by the addition of water (6 mL), and the mixture was filtered through a small frit (D3) to give 1-benzyl-4-phenyl-1,2,3-triazole (**9**) as a pale yellow solid (89 mg, 75 % isolated yield) with identical physical properties as reported above.

**Crystal Structure Determination:** The crystal structures were determined with a Bruker APEX-II CCD diffractometer by using graphite-monochromatized Mo-*K*_α_ radiation (0.71073 Å). Structures were solved by direct methods and refined by using the SHELXL and SHELXS suite of programs.^53^ Further details are given in the Supporting Information. Structural and refinement data for **2**, **3**, **4a**, **4b**, and **4c** are given in Table 2. CCDC-CCDC-795562 (for **2**), and-CCDC-771838 (for **3**), -CCDC-771839 (for **4a**), -CCDC-771840 (for **4b**), and -CCDC-771841 (for **4c**) contain the supplementary crystallographic data for this paper. These data can be obtained free of charge from The Cambridge Crystallographic Data Centre via http://www.ccdc.cam.ac.uk/data_request/cif.

**Table 2:** Summary of structural and refinement data of **2**, **3**, **4a**, **4b**, and **4c**

	**2**	**3**	**4a**	**4b**	**4c**
Empirical formula	C_14_H_20_F_5_N_2_PSe	C_28_H_40_Cl_2_F_10_N_4_P_2_Pd	C_28_H_40_CuF_10_IN_4_P_2_	C_28_H_40_CuF_10_IN_4_P_2_	2**·**(C_28_H_40_CuF_10_IN_4_P_2_)**·**C_4_H_8_O
Formula mass	421.25	861.88	875.02	875.02	1822.14
Crystal description	needle, colorless	plate, orange	needle, colorless	plate, colorless	plate, colorless
Crystal size [mm]	0.62 × 0.10 × 0.09	0.298 × 0.192 × 0.068	0.34 × 0.12 × 0.10	0.35 × 0.30 × 0.16	0.38 × 0.34 × 0.13
Crystal system	orthorhombic	monoclinic	monoclinic	monoclinic	monoclinic
Space group	*Pca*2_1_	*I*2/*a*	*P*2_1_/*c*	*Ia*	*P*2/*c*
Unit cell dimensions					
*a* [Å]	17.5973(5)	24.1455(10)	16.8291(7)	18.8155(11)	25.4888(9)
*b* [Å]	7.0871(2)	9.0003(4)	11.6826(5)	10.0910(4)	18.3825(6)
*c* [Å]	27.5772(7)	48.655(2)	18.7734(7)	20.5851(8)	16.3908(6)
*β* [°]	90	92.825(2)	104.779(2)	115.0890(10)	100.3930(10)
Volume [Å^3^]	3439.26(16)	10560.8(8)	3568.9(3)	3539.7(3)	7553.9(5)
*Z*	8	12	4	4	4
Calculated density [Mg m^–3^]	1.627	1.626	1.629	1.642	1.602
*F*(000)	1696	5232	1752	1752	3664
*μ* [mm^–1^]	2.324	0.850	1.644	1.658	1.558
Absorption correction	semiempirical from equivalents				
Temperature [K]	100	100	100	100	100
Θ range for data collection [°]	2.31–26.00	1.85–30.00	2.07–30.00	2.18–35.00	1.96–30.00
Index ranges	–21 ≤ *h* ≤ 21	–33 ≤ *h* ≤ 32	23 ≤ *h* ≤ 23	–30 ≤ *h* ≤ 30	–35 ≤ *h* ≤ 35
	–8 ≤ *k* ≤ 8	–12 ≤ *k* ≤ 12	–16 ≤ *k* ≤ 16	–16 ≤ *k* ≤ 16	–24 ≤ *k* ≤ 25
	–33 ≤ *l* ≤ 34	–68 ≤ *l* ≤ 67	–26 ≤ *l* ≤ 26	–33 ≤ *l* ≤ 33	–21 ≤ *l* ≤ 23
Reflections collected/unique	46642/6691	113786/15338	105420/10398	31479/13206	135615/21718
Reflections observed [*I* > 2σ(*I*)]	6392	12368	9069	12829	18627
*R*(int), *R*(sigma)	0.0410, 0.0316	0.0403, 0.0284	0.0350, 0.0185	0.0199, 0.0335	0.0279, 0.0198
Data/restraints/parameters	6691/428/14	15338/737/36	10398/480/0	13206/520/16	21718/1122/368
Goodness-of-fit on *F*^2^	1.187	1.034	1.038	1.007	1.273
*R*_1_, *wR*_2_ [*I* > 2σ(*I*)]	0.0559, 0.1378	0.0344, 0.0845	0.0208, 0.0496	0.0162, 0.0382	0.0357, 0.0720
*R*_1_, *wR*_2_ (all data)	0.0588, 0.1390	0.0490, 0.0935	0.0277, 0.0530	0.0170, 0.0385	0.0447, 0.0753
Largest difference peak/hole [e Å^–3^]	2.330/–0.998	1.419/–1.089	0.805/–0.549	0.725/–0.390	1.047/–1.534

**Supporting Information** (see footnote on the first page of this article): Further experimental details including spectra, crystallographic information, information on catalytic studies, as well as computational details.
