# Supplementary material for: Bis(diethylamino)(pentafluorophenyl)phosphane – a Push–Pull Phosphane Available for Coordination
Source: Eur J Inorg Chem. 2011 Apr 27;2011(16):2588–96. doi: 10.1002/ejic.201100138 (PMC3318678; doi:10.1002/ejic.201100138)
Supplement: Supplementary file 1 [file ejic2011-2588-SD1.pdf]

Eur. J. Inorg. Chem. **2011** · © WILEY-VCH Verlag GmbH & Co. KGaA, 69451 Weinheim, 2011 · ISSN 1434–1948

SUPPORTING INFORMATION

DOI: 10.1002/ejic.201100138
Title: Bis(diethylamino)(pentafluorophenyl)phosphane – a Push–Pull Phosphane Available for Coordination
Author(s): Andreas Orthaber, Michael Fuchs, Ferdinand Belaj, Gerald N. Rechberger, C. Oliver Kappe, Rudolf Pietschnig*

Experimental Section

MS data of ligand (2) and complexes (3) and (4)

EI-MS measurements of ligand **2**: [m/z] 422.1 (41%, $C_6F_5P(Se)(NEt_2)_2^+$), 341.1 (57%, M^+-Se), 350.1 (13%, M^+-NEt_2), 270.1 (100%, M^+-Se-NEt_2), 111.0 (36%, PSe^+), 72.1 (100%, NEt_2^+). The typical pattern of compound 2 is displayed in the subsequent figure.

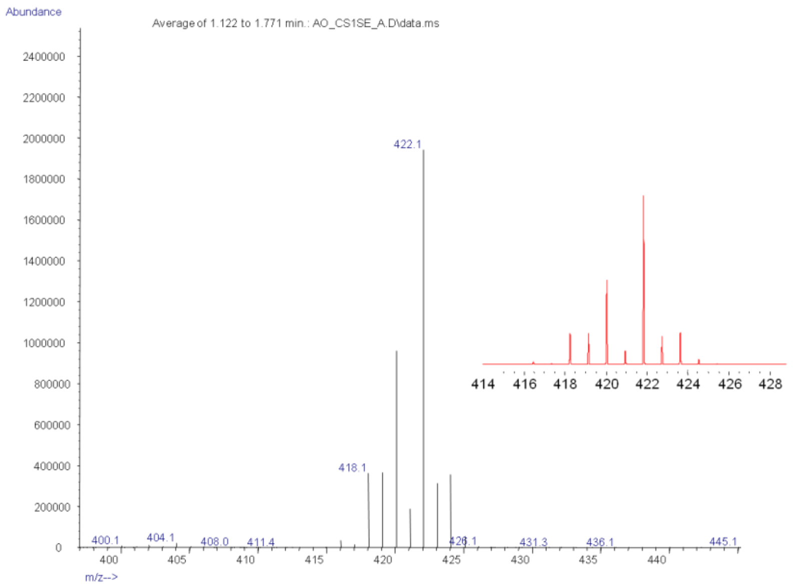

Figure 1: Expansion of the MS spectra in the M^+ region for ligand **2**. Insert on the right hand side shows the matching calculated isotopic distribution for $C_{14}H_{20}F_5PN_2Se$.

	calc. [m/z]	found [m/z]	Δ [mDa]	Δ [ppm]
$[C_6F_5P(NEt_2)_2 H]^+$	343.1371	343.1363	0.80	2.3
$[C_6F_5P]^+$	270.0486	270.0471	1.50	5.6
$[C_6F_5P H]^+$	198.9746	198.9736	1.00	5.0

Table 1: Relevant HR-MS data of fragments found for complex **3**.

	calc. [m/z].	found [m/z]	Δ [mDa]	Δ [ppm]
[3*Cu]+	937.0409	937.0405	0.40	0.4
[3-I]+	747.1906	747.1964	5.80	7.8
[C6F5P*H]+	270.0473	270.0471	0.20	0.7

Table 2: Relevant HR-MS data of complex **4** and its fragments.

NMR-Spectra of compounds **1**, **2**, **3**, and **4**

<u>Bis(diethylamino)-pentafluorophenyl phosphane (**1**)</u>

$^{1}H(C_6D_6$,300 MHz, 298K)

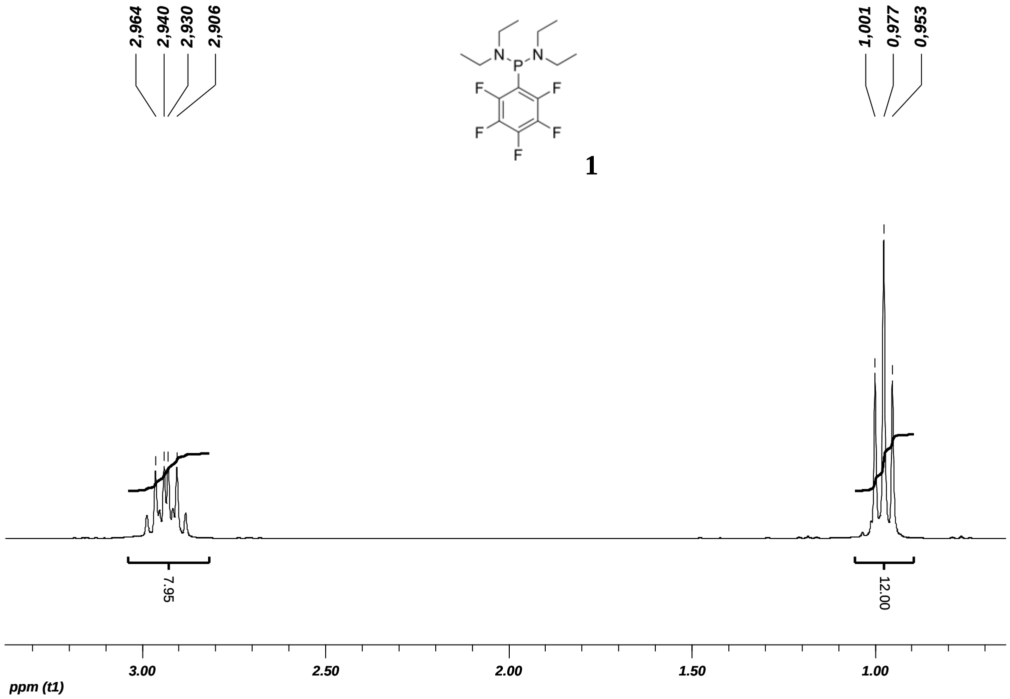

$^{13}C(C_6D_6$, 75.5MHz, 298K)

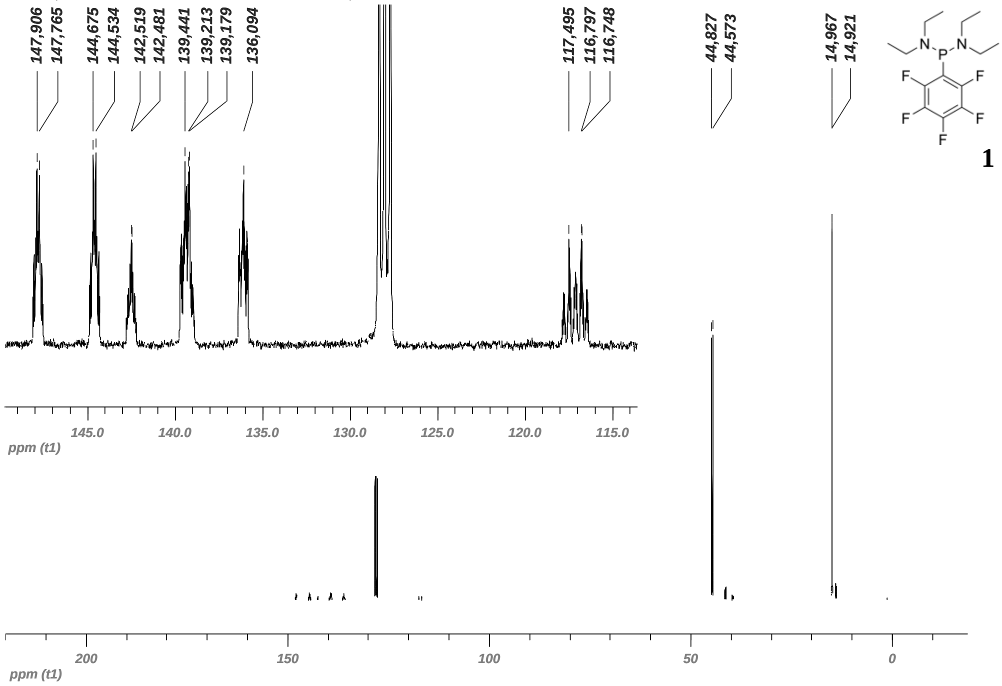

^{19}F(C_6D_6, 282.4MHz, 298K)

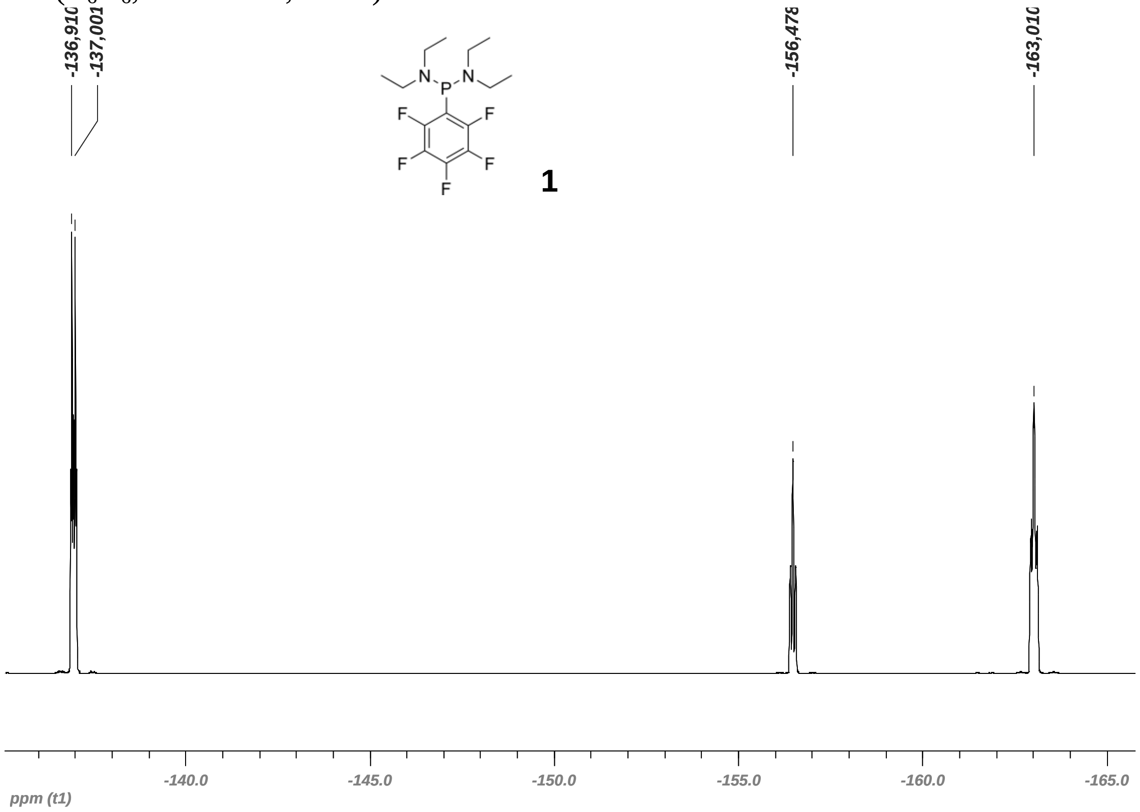

Bis(diethylamino)(pentafluorophenyl)selenophosphorane]palladium (**2**)

$^{1}H(CDCl_3$,400 MHz, 298K)

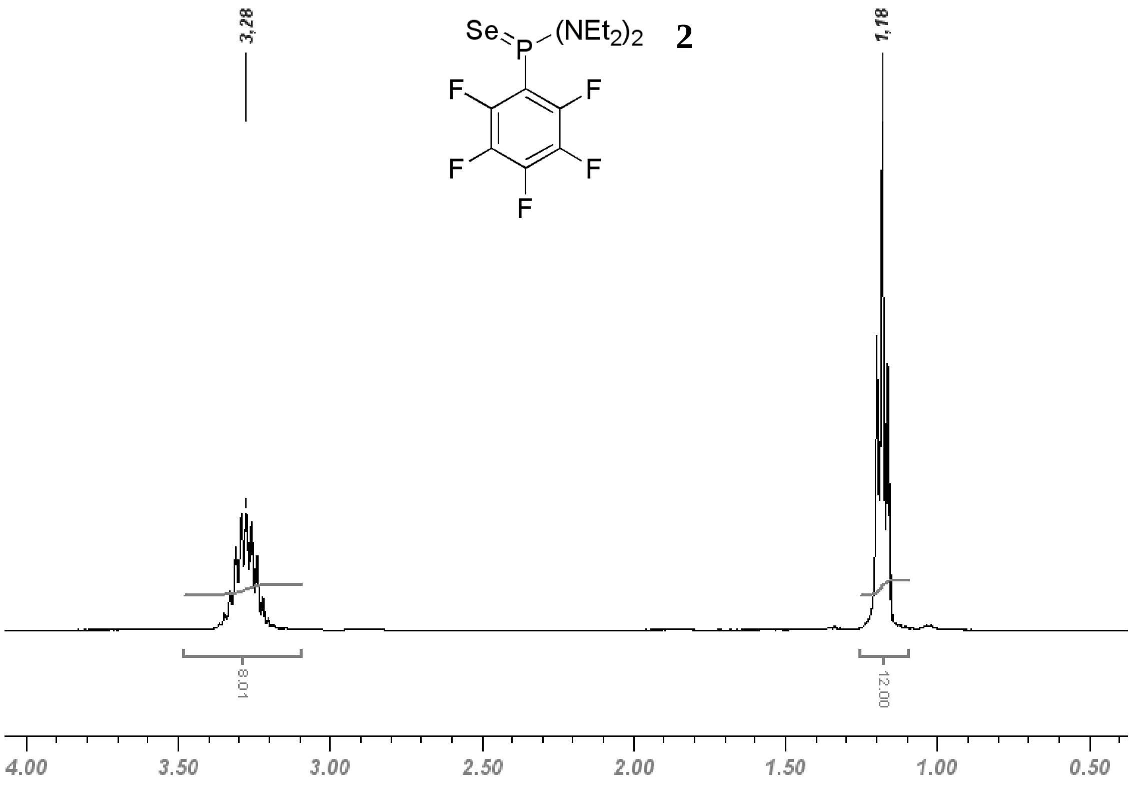

$^{13}C(CDCl_3$,100 MHz, 298K)

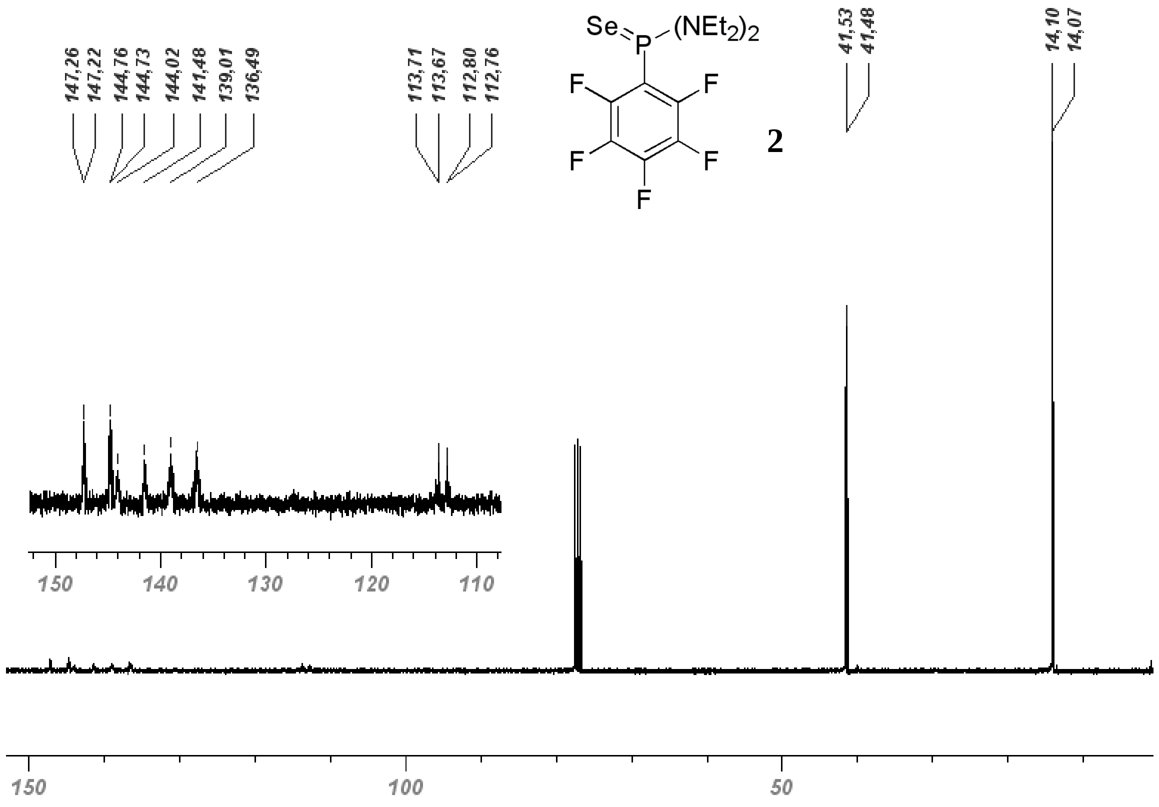

$^{31}P(CDCl_3$,145 MHz, 298K)

2

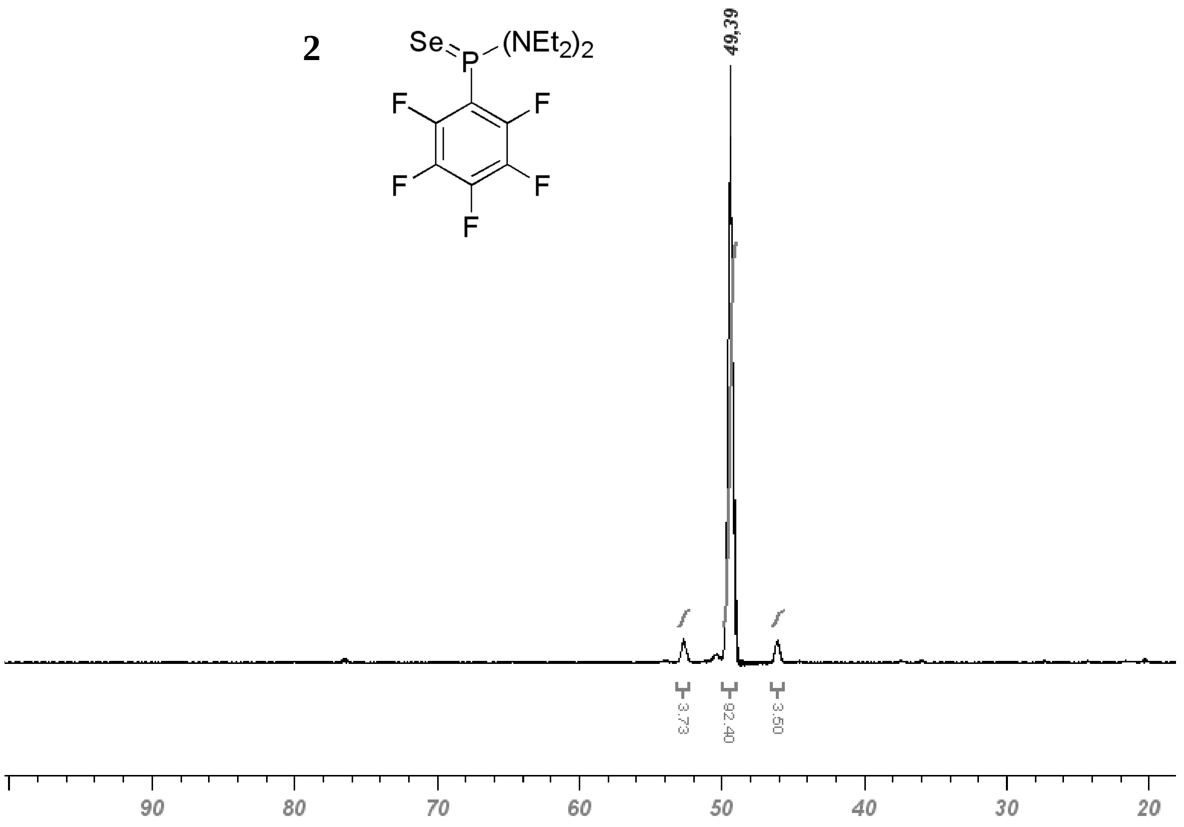

$^{19}F(CDCl_3$,145 MHz, 298K)

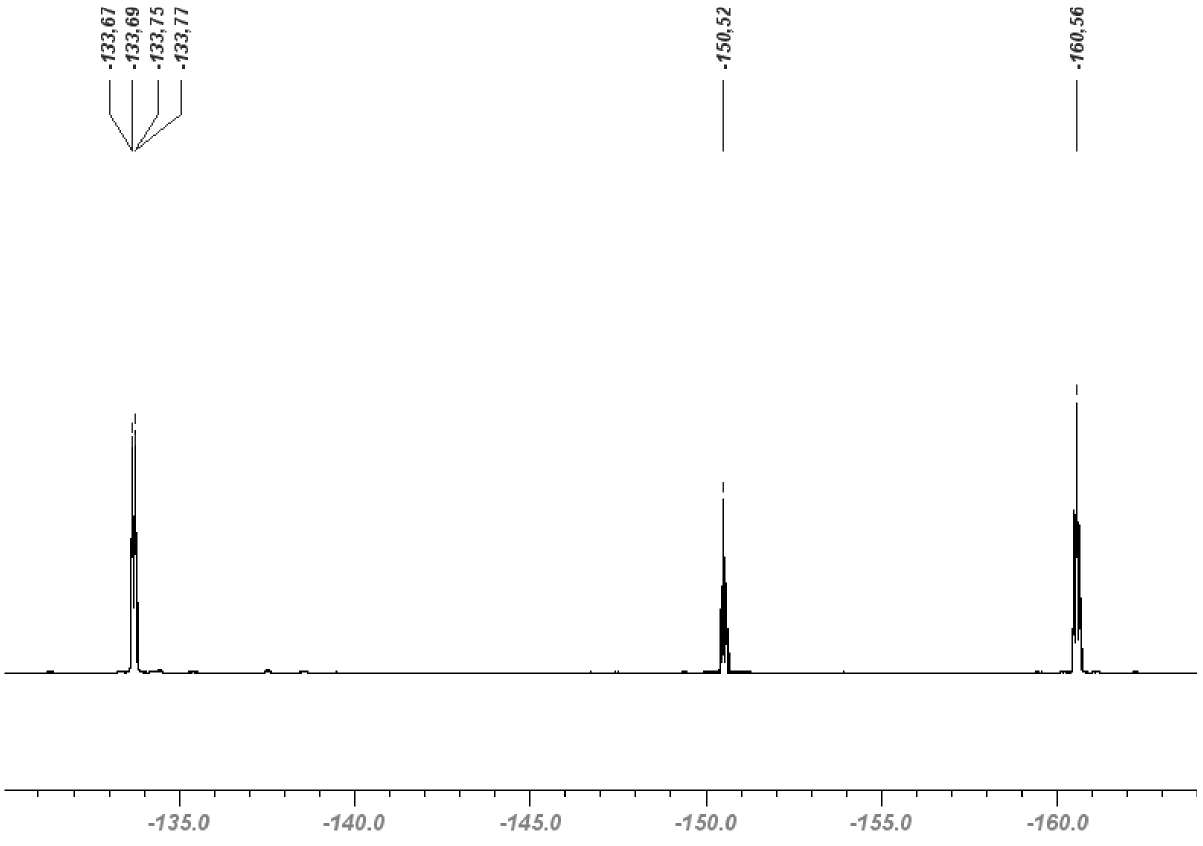

^{77}Se ($CDCl_3$, 57.2 MHz, 298K)

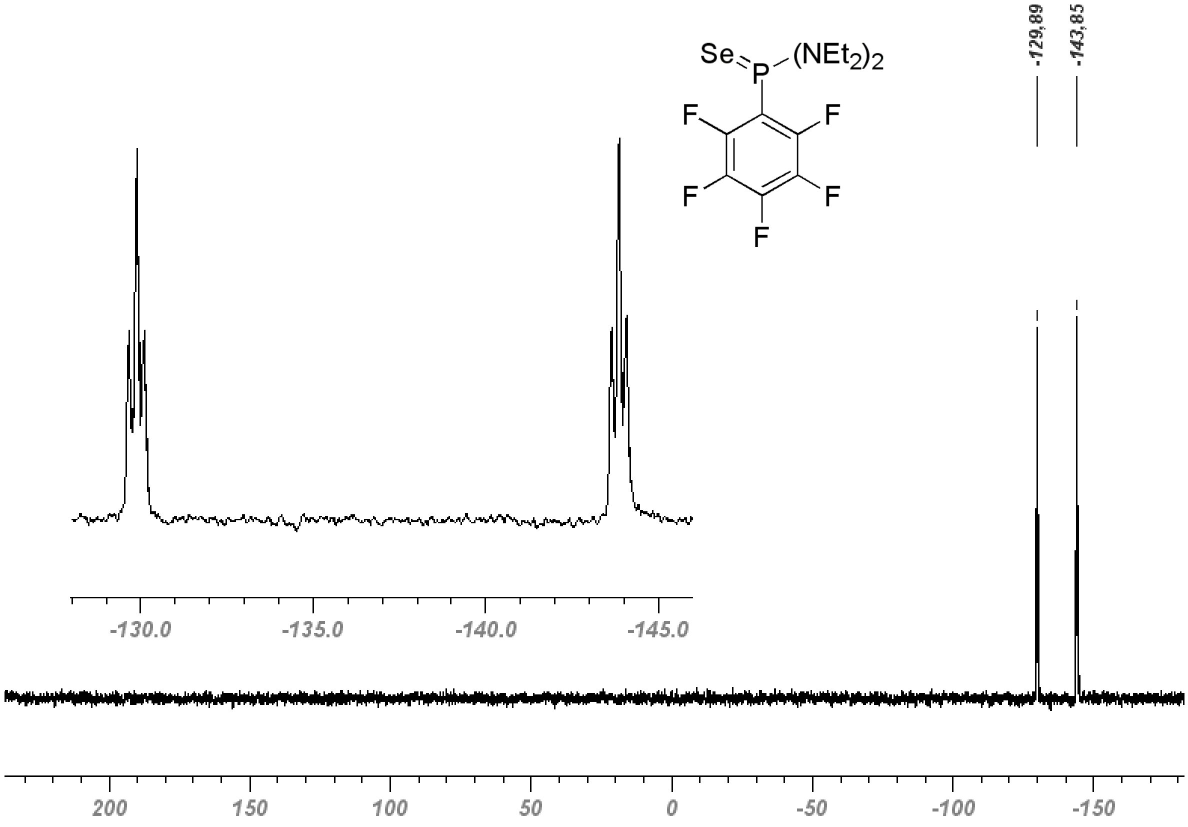

<u>*trans*-dichloro-bis[bis(diethylamino)(pentafluorophenyl)phosphane]palladium (**3**)</u>

^{1}H(C_6D_6,300 MHz, 298K); * MeCN

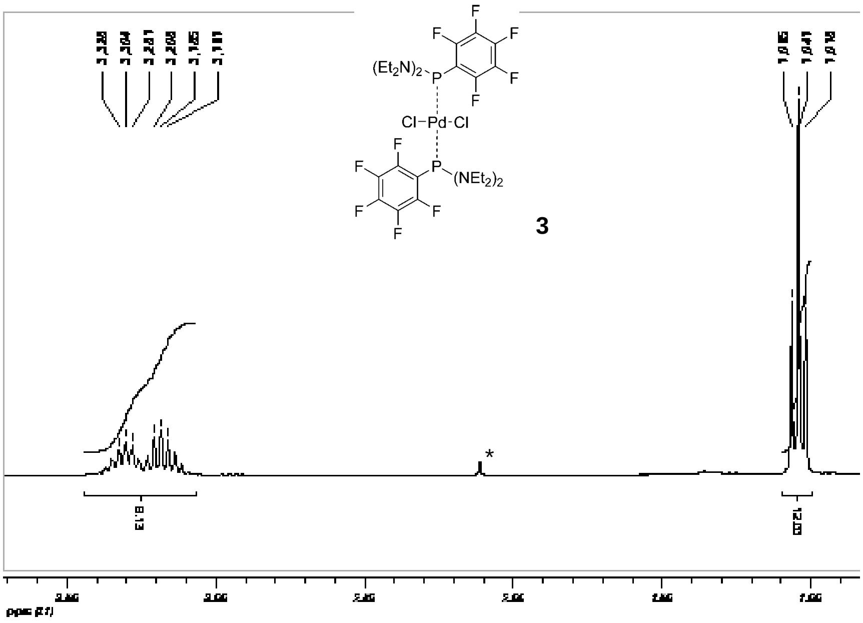

^{13}C(C_6D_6, 75.5MHz, 298K)

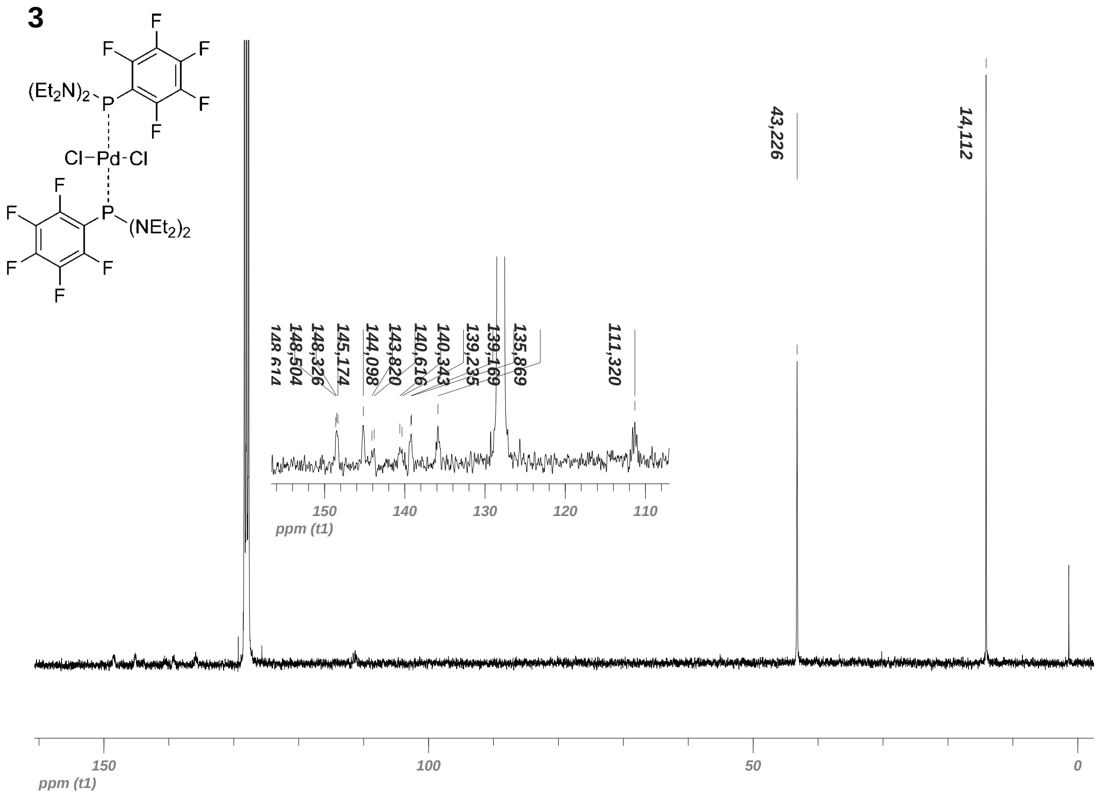

$^{19}F(C_6D_6$, 282.4MHz, 298K)

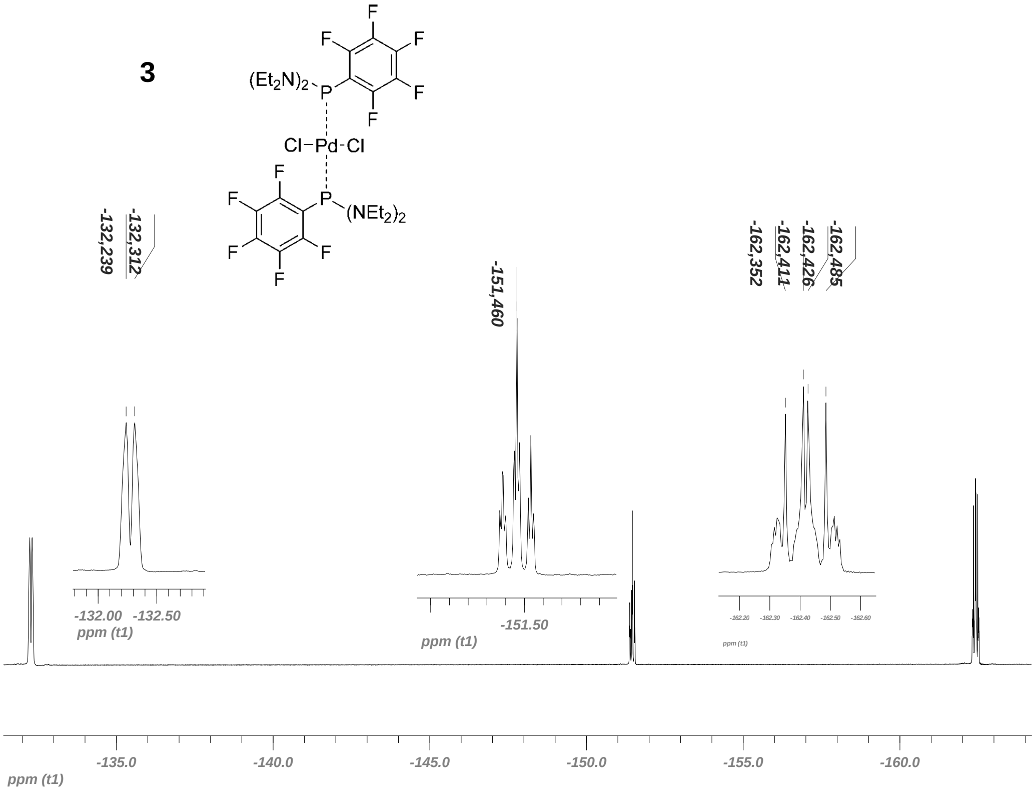

iodo-bis[bis(diethylamino)(perfluorophenyl)phosphine]copper (**4**)

^{1}H(CDCl$_3$,300 MHz, 298K)

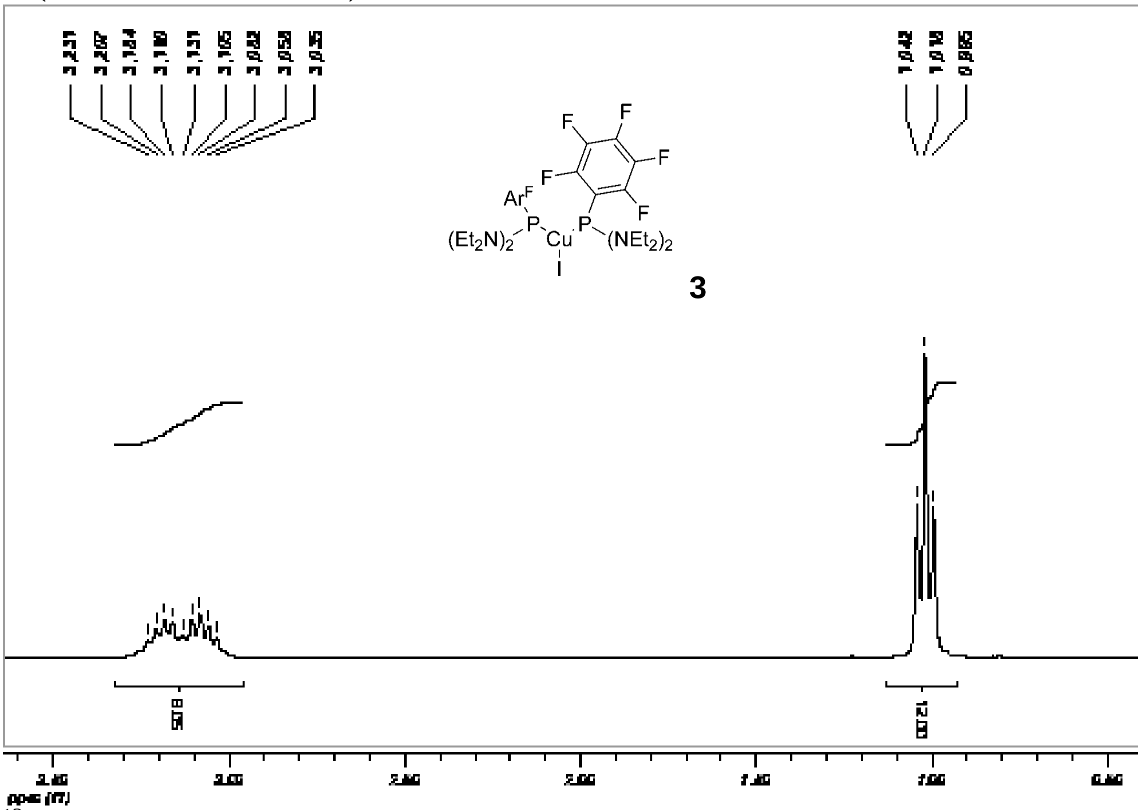

^{13}C(CDCl$_3$, 100.6 MHz, 298K)

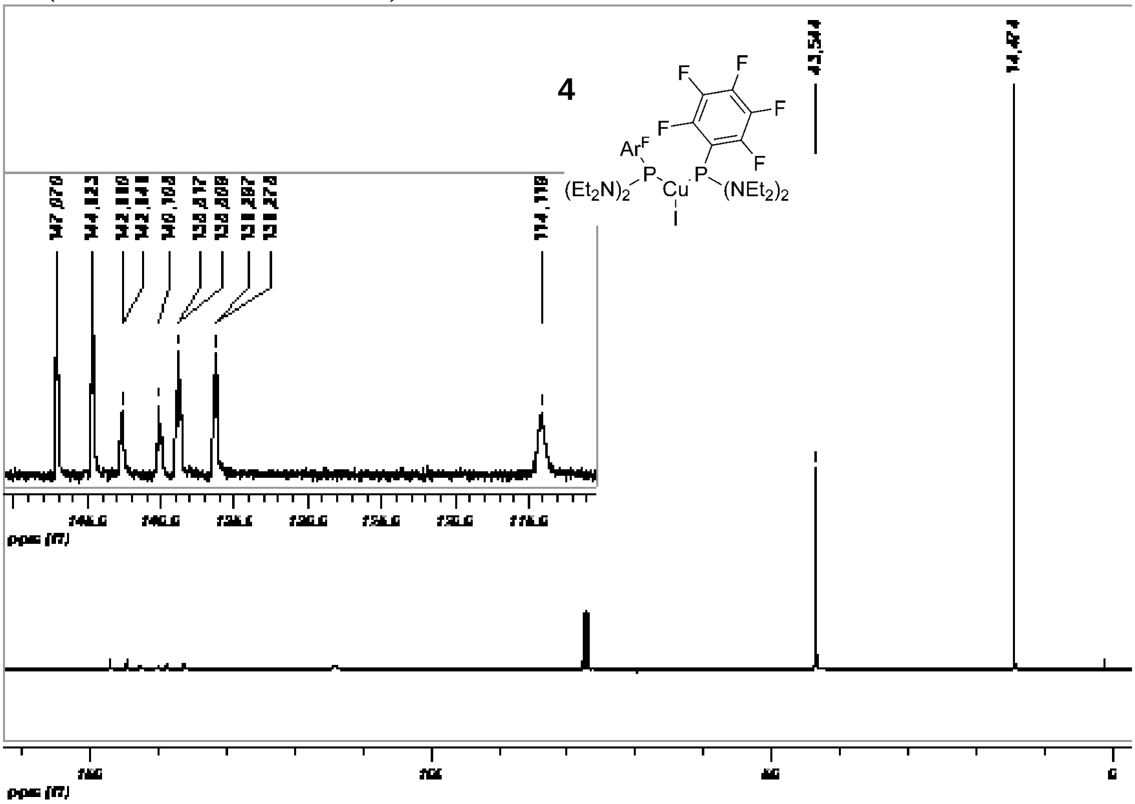

^{19}F(CDCl$_3$, 282.4MHz, 298K)

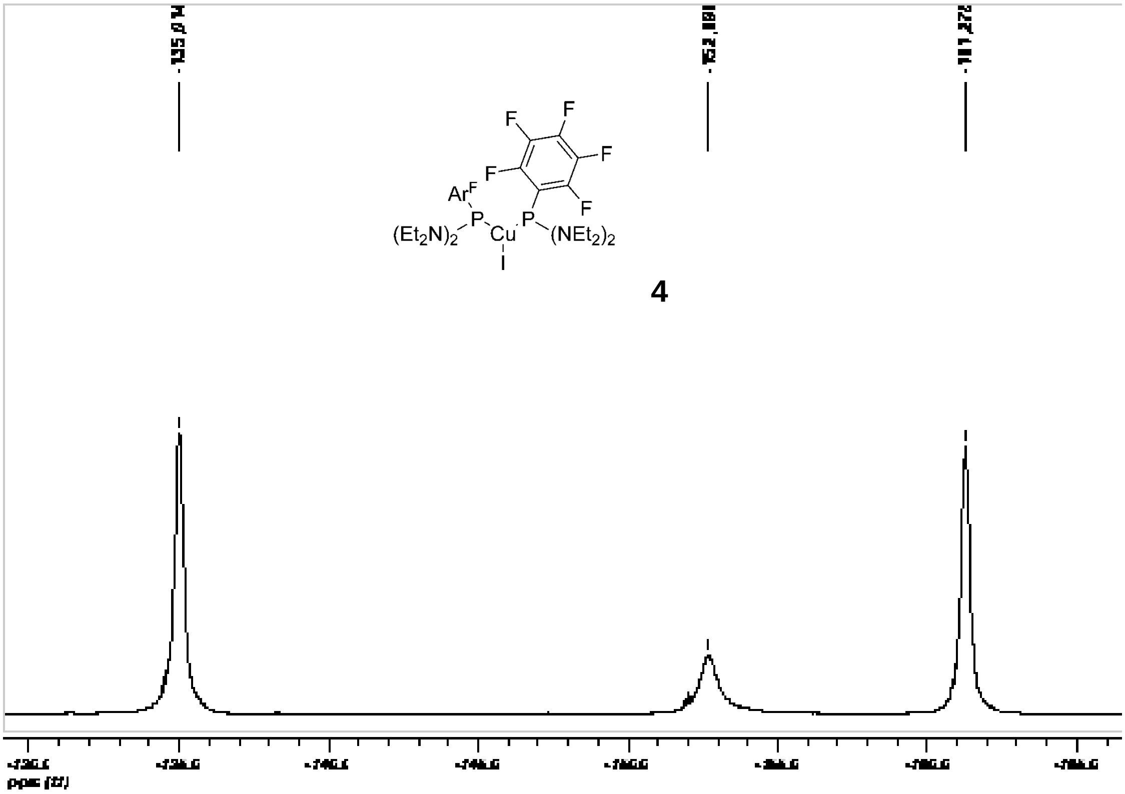
F
F
F
F
F
Ar^F
P
P
(Et2N)2
Cu
(NEt2)2
I
4

DFT calculations

All compounds have been fully optimized without any constraints at the B3LYP[1, 2] level of theory. All atoms have been treated with the 6-31G* basis set, except Pd where an LANL2DZ[3, 4] ECP basis set was used. Structures are true minima on the PES (no imaginary frequency). Thermal correction was calculated at 298 K, and no scaling factor was applied for harmonic frequency correction. Calculations were carried out with the Gaussian G03 (Rev. B.04) program package[5] on a linux cluster (Fedora Core 3).

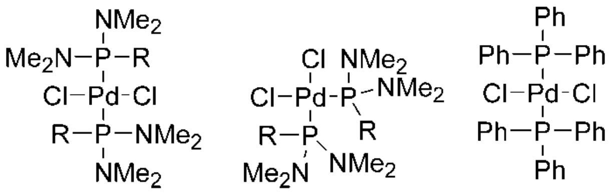

trans-**M1** *cis*-**M1** **M2**

Scheme 1: Model compounds **M1** (R= C_6F_5) and **M2**. Structures of **M1** have been optimized for both *cis* and *trans*-configuration.

	cis-**M1**	*trans*-**M1**
ΔE_{elec}	12.8	0.0
$\Delta E_{elec+ZPV}$	13.6	0.0
ΔH	13.2	0.0
ΔG^{298}	15.3	0.0

Table 1: summary of energy differences for *cis* and *trans*-**M1.** The energetically favoured isomer (*trans*-**M1**) is arbitrarily set to 0!. Energy differences are given in [kcal mol^{-1}].

trans-**M1**

number	atom	x	y	z
1	C	-5.605436	-0.756705	0.469922
2	C	-5.685552	-1.238644	-0.832386
3	C	-4.696954	-0.902201	-1.754393
4	C	-3.643589	-0.091338	-1.352318
5	C	-3.528208	0.421756	-0.055072
6	C	-4.534760	0.055532	0.840568
7	F	-2.706427	0.202454	-2.269671
8	F	-4.765107	-1.365876	-3.006643
9	P	-1.977667	1.367821	0.375982
10	Pd	-0.003809	-0.019768	0.267274
11	P	1.962424	-1.347804	0.172272
12	N	2.259354	-2.205259	1.615511
13	C	2.350203	-1.391608	2.835497
14	H	1.356515	-1.210069	3.271166
15	F	-6.706632	-2.015256	-1.198601
16	F	-4.526789	0.480361	2.118569
17	F	-6.556314	-1.069921	1.357849
18	C	1.487964	-3.436209	1.840435
19	H	2.009802	-4.026788	2.602746
20	C	3.515128	-0.342605	-0.075768
21	C	3.468826	0.605065	-1.106319
22	C	4.546792	1.413010	-1.443665
23	C	5.743871	1.273280	-0.745086
24	C	5.842901	0.323406	0.265727
25	C	4.743226	-0.476113	0.579951
26	F	6.794173	2.033596	-1.056640
27	F	4.448202	2.307526	-2.431864
28	F	6.997539	0.167712	0.923296
29	F	2.347084	0.742064	-1.835526
30	F	4.935158	-1.385032	1.548512
31	N	2.067299	-2.543832	-1.014282
32	C	1.360862	-2.393270	-2.289789
33	H	1.151571	-3.391252	-2.691841
34	C	3.313421	-3.308765	-1.124389
35	H	4.026103	-2.834437	-1.816932
36	N	-2.062166	2.648419	-0.721369
37	C	-3.306746	3.157194	-1.294978
38	H	-3.259334	4.252818	-1.347912
39	C	-0.879099	3.071986	-1.476801
40	H	-0.928967	2.705028	-2.512769
41	N	-2.144433	2.029635	1.913218
42	C	-3.010137	3.165753	2.203971
43	H	-2.566243	3.747115	3.020727
44	C	-1.709266	1.270755	3.083586
45	H	-2.552754	0.779294	3.587017
46	Cl	1.292079	1.823512	1.078528
47	Cl	-1.282096	-1.996510	-0.098908
48	H	3.785786	-3.408405	-0.145032
49	H	3.082420	-4.311534	-1.502828
50	H	1.961993	-1.839806	-3.026889
51	H	0.409321	-1.885823	-2.136915
52	H	1.422648	-4.017907	0.921211
53	H	-3.465664	2.772124	-2.312972
54	H	2.966097	-1.922159	3.569929
55	H	2.807546	-0.422016	2.633815
56	H	0.033015	2.704407	-1.008965
57	H	-0.834332	4.168442	-1.498570
58	H	0.466369	-3.226493	2.186732
59	H	-4.168602	2.884787	-0.683961
60	H	-0.984797	0.509490	2.783668
61	H	-3.078274	3.818753	1.331951
62	H	-1.215239	1.948211	3.790052
63	H	-4.019888	2.853170	2.507395

Table 2: Coordinates (xyz) of *trans*-**M1**.

E(RB+HF-LYP)	-3723.79469
Zero-point correction	0.44232
Thermal correction to Energy	0.48862
Thermal correction to Enthalpy	0.48957
Thermal correction to Gibbs Free Energy	0.36005
Sum of electronic and zero-point Energies	-3723.35237
Sum of electronic and thermal Energies	-3723.30607
Sum of electronic and thermal Enthalpies	-3723.30513
Sum of electronic and thermal Free Energies	-3723.43464

Table 3: Energy output for *trans*-**M1**.

cis-**M1**

number	atom	x	y	z
1	C	5.435033	-0.994483	-0.686303
2	C	5.313003	-2.105839	0.141182
3	C	4.216379	-2.211869	0.993876
4	C	3.263211	-1.201789	0.997556
5	C	3.349262	-0.067636	0.184908
6	C	4.45801	-0.001761	-0.661103
7	F	2.220329	-1.340088	1.836728
8	F	4.094495	-3.268795	1.802173
9	P	1.906123	1.114168	0.122524
10	Pd	0.053139	-0.163151	-0.697946
11	P	-1.731609	0.683529	0.601747
12	N	-1.401684	0.47852	2.247514
13	C	-0.734065	-0.753482	2.682899
14	H	-0.015904	-0.51015	3.474679
15	F	6.244859	-3.058285	0.127521
16	F	4.630448	1.023372	-1.511306
17	F	6.483846	-0.886258	-1.507483
18	C	-2.137932	1.161894	3.307505
19	H	-3.088065	0.665718	3.551062
20	C	-3.395869	-0.080222	0.219538
21	C	-3.96445	0.178733	-1.033291
22	C	-5.155713	-0.393412	-1.45816
23	C	-5.825222	-1.275754	-0.612815
24	C	-5.294661	-1.562712	0.639986
25	C	-4.096231	-0.970265	1.034308
26	F	-6.973384	-1.832485	-0.998513
27	F	-5.657842	-0.110966	-2.663492
28	F	-5.937186	-2.3981	1.463511
29	F	-3.346944	1.01522	-1.885896
30	F	-3.648607	-1.301677	2.260819
31	N	-2.083289	2.343232	0.407592
32	C	-1.42494	3.111635	-0.655082
33	H	-1.310976	4.152164	-0.325515
34	C	-3.409789	2.893623	0.709868
35	H	-4.034016	2.952315	-0.193107
36	N	1.97255	1.752701	1.695786
37	C	3.087429	1.659022	2.636801
38	H	3.540877	2.648758	2.801383
39	C	1.012509	2.780472	2.09251
40	H	0.72469	2.623747	3.139163
41	N	2.223042	2.44089	-0.877562
42	C	3.228516	3.447258	-0.533595
43	H	2.851466	4.439332	-0.811575
44	C	1.87572	2.435072	-2.302463
45	H	2.729887	2.132209	-2.91997
46	Cl	-1.446796	-1.813448	-1.522747
47	Cl	1.656378	-1.005589	-2.261672
48	H	-3.935139	2.290537	1.450733
49	H	-3.294308	3.908105	1.112166
50	H	-2.014172	3.099404	-1.581576
51	H	-0.435558	2.709824	-0.866363
52	H	-2.325763	2.201587	3.033583
53	H	2.730813	1.285544	3.605626
54	H	-1.449365	-1.491918	3.063175
55	H	-0.185913	-1.197342	1.850393
56	H	0.105032	2.735981	1.494429
57	H	1.447132	3.788588	2.004303
58	H	-1.516873	1.165046	4.210753
59	H	3.864157	0.989117	2.274232
60	H	1.061892	1.735614	-2.496024
61	H	3.421542	3.445147	0.540423
62	H	1.564128	3.446564	-2.592421
63	H	4.173772	3.271466	-1.061612

Table 4: Coordinates (xyz) of *cis*-**M1**

E(RB+HF-LYP)	-3723.77434
Zero-point correction	0.44362
Thermal correction to Energy	0.48928
Thermal correction to Enthalpy	0.49022
Thermal correction to Gibbs Free Energy	0.36408
Sum of electronic and zero-point Energies	-3723.33071
Sum of electronic and thermal Energies	-3723.28506
Sum of electronic and thermal Enthalpies	-3723.28412
Sum of electronic and thermal Free Energies	-3723.41025

Table 5: Energy output for *cis*-**M1**.

trans-**M2**

number	atom	x	y	z
1	C	4.231407	-0.499885	-4.400306
2	C	3.749359	0.688510	-3.846044
3	C	4.151003	-1.685617	-3.670636
4	C	3.187189	0.691253	-2.571120
5	C	3.598256	-1.686015	-2.387774
6	C	-3.602040	-2.359286	-2.597954
7	C	-4.909962	-2.646375	-2.203697
8	C	-4.328922	3.599254	-1.703193
9	C	3.115818	-0.496697	-1.822646
10	C	-3.429861	4.653416	-1.533844
11	C	-2.837137	-1.433911	-1.886013
12	C	-5.456119	-2.001388	-1.092374
13	C	-4.017148	2.326311	-1.222135
14	C	-2.211569	4.431416	-0.887866
15	C	-3.378882	-0.781643	-0.766562
16	C	-4.696777	-1.075377	-0.375628
17	C	-2.797124	2.097717	-0.570931
18	C	-1.892034	3.158562	-0.416032
19	C	1.892034	-3.158562	0.416032
20	C	4.696777	1.075377	0.375628
21	C	2.797124	-2.097717	0.570931
22	C	2.211569	-4.431416	0.887866
23	C	3.378882	0.781643	0.766562
24	C	5.456119	2.001388	1.092374
25	C	4.017148	-2.326311	1.222135
26	C	3.429861	-4.653416	1.533844
27	C	4.328922	-3.599254	1.703193
28	C	2.837137	1.433911	1.886013
29	C	-3.115818	0.496697	1.822646
30	C	4.909962	2.646375	2.203697
31	C	3.602040	2.359286	2.597954
32	C	-3.598256	1.686015	2.387774
33	C	-3.187189	-0.691253	2.571120
34	C	-4.151003	1.685617	3.670636
35	C	-3.749359	-0.688510	3.846044
36	C	-4.231407	0.499885	4.400306
37	H	4.663850	-0.500444	-5.397370
38	H	3.798899	1.614914	-4.411530
39	H	4.522725	-2.615011	-4.093815
40	H	-3.170479	-2.856285	-3.462517
41	H	-5.501271	-3.370273	-2.758290
42	H	-5.274207	3.764326	-2.213259
43	H	2.799421	1.616930	-2.160273
44	H	-3.673931	5.643099	-1.910732
45	H	3.550470	-2.614411	-1.829198
46	H	-1.826911	-1.201910	-2.206136
47	H	-4.721529	1.512481	-1.362404
48	H	-6.473397	-2.219598	-0.778316
49	H	-1.502032	5.244957	-0.764356
50	H	5.128255	0.588353	-0.492718
51	H	0.931217	-2.981157	-0.058129
52	H	-5.128255	-0.588353	0.492718
53	H	-0.931217	2.981157	0.058129
54	H	1.502032	-5.244957	0.764356
55	H	6.473397	2.219598	0.778316
56	H	4.721529	-1.512481	1.362404
57	H	3.673931	-5.643099	1.910732
58	H	-3.550470	2.614411	1.829198
59	H	1.826911	1.201910	2.206136
60	H	5.274207	-3.764326	2.213259
61	H	-2.799421	-1.616930	2.160273
62	H	5.501271	3.370273	2.758290
63	H	3.170479	2.856285	3.462517
64	H	-4.522725	2.615011	4.093815
65	H	-3.798899	-1.614914	4.411530
66	H	-4.663850	0.500444	5.397370
67	Cl	-0.081156	0.757317	-2.248738
68	Cl	0.081156	-0.757317	2.248738
69	P	2.362145	-0.459470	-0.142920
70	P	-2.362145	0.459470	0.142920
71	Pd	0.000000	0.000000	0.000000

Table 6: Coordinates (xyz) of *trans*-**M2**

E(RB+HF-LYP)	-3119.83612
Zero-point correction	0.55504
Thermal correction to Energy	0.59416
Thermal correction to Enthalpy	0.59511
Thermal correction to Gibbs Free Energy	0.47587
Sum of electronic and zero-point Energies	-3119.28109
Sum of electronic and thermal Energies	-3119.24196
Sum of electronic and thermal Enthalpies	-3119.24101
Sum of electronic and thermal Free Energies	-3119.36025

Table 7: Energy output for *trans*-**M2**.

Selected MOs:of *trans*-**M1** at an isolevel of 0.09 a.u.

Orbital 173(LUMO)

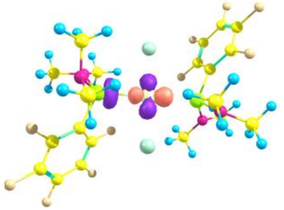

Orbital 165(HOMO-7))

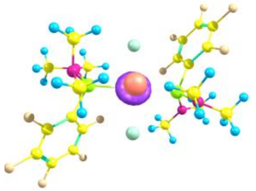

Orbital 163(HOMO-9) Orbital 161(HOMO-11)

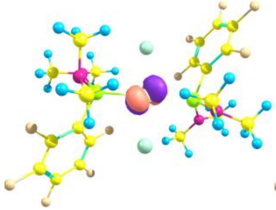

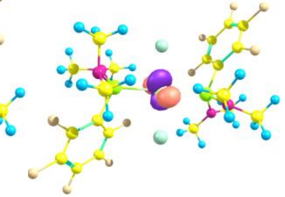

Orbital 157(HOMO-15)

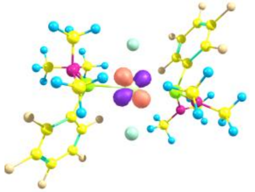

The d-orbitals at the Pd center for a square planar complex are altered with respect to their order. The HOMO-7 is the Pd(dz^2) orbital and the Pd(dxz), Pd(dyz) orbitals are almost degenerated (H-9,H-11), and the lowest orbital in this series is the Pd(dxy) (H-15). A detailed summary of the orbital energies, and relevant occupation factors is given in table 8 and 9. Comparison of *trans*-**M1** with the bis-PPh_3 substituted palladium complex shows that the order of the d-orbitals remains the same, but the LUMO and the Pd(dz^2) are stabilized (-0.0797 and -0.2347 in **M2**) in *trans*-**M1** with respect to **M2**

Orbital	Type[a]	Energy[b]	Major contributions from AOs
LUMO	$d(x^2-y^2)$	-0.1796	**Pd(7d-2) -0.67739;** P(4pz) 0.52685
HOMO-7	$d(z^2)$	-0.2794	**Pd(7d+0) 0.74539**, P(d7d+1) -0.35797
HOMO-9	d(xz)	-0.2863	**Pd(7d+1) 0.82682**, Pd(8d+1) 0.25499
HOMO-11	d(yz)	-0.2869	**Pd(7d-1) 0.75706**, Pd(7d0) 0.36987
HOMO-15	d(xy)	-0.3117	**Pd(7d+2) -0.78982**, Pd(7d-2) -0.30128

Table 8: Selected Palladium d-orbitals of *trans*- **M1** [a] by visual inspection of the orbital plots . x and y axis are exchangeable. [b] in hartree.

.

Selected MOs:of *trans*-**M2** at an isolevel of 0.09 a.u.

Orbital 165(LUMO)

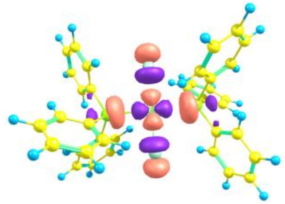

Orbital 161(HOMO-3)

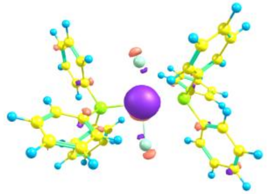

Orbital 146(HOMO-18)

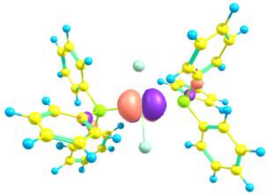

Orbital 145(HOMO-19)

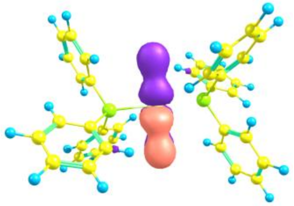

Orbital 141(HOMO-23)

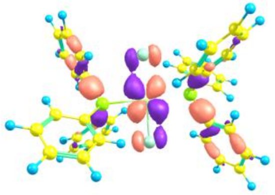

Orbital	Type[a]	Energy[b]	Major contributions from AOs
LUMO	$d(x^2\text{-}y^2)$	-0.0797	**Pd(7d0) 0.48965**, Pd(7d+2) 0.29967
HOMO-3	$d(z^2)$	-0.2347	**Pd(7d+2) 0.41609**, Pd(7d-1) -0.29861
HOMO-18	d(xz)	-0.2780	**Pd(7d-2) 0.67348,** Pd(7d+2) 0.28505
HOMO-19	d(yz)	-0.3008	**Pd(7d-1) 0.49112,** Pd(7d0) 0.32130
HOMO-23	d(xy)	-0.3125	**Pd(7d+1) 0.50006**

Table 9: Selected Palladium d-orbitals of *trans*- **M2** [a] by visual inspection of the orbital plots . x and y axis are exchangeable. [b] in hartree.

Details of the crystal structure determinations of 2, 3 and 4a-c

Crystallographic data for 2: $C_{14}H_{20}F_5N_2PSe$, M_r=421.25, orthorhombic space group P c a 2_1, a=17.5973(5) Å, b= 7.0871(2) Å, c=27.5772(7) Å, V=3439.26(16) Å^3, Z=8, d_{calc}=1.627 g·cm^{-3}, $\mu(Mo_{K\alpha})$=0.850 mm^{-1}, T=100 K, F(000)=1696, 46642 reflections measured, 6691 unique (R_{int}=0.0410), data/parameters/restraints= 6691/428/14, R1=0.0559 ($I > 2\sigma(I)$), wR2=0.1378 (all), Flack= 0.348(13), GOF=1.187. The asymmetric unit contains two independent molecules. All non-hydrogen atoms were refined with anisotropic displacement parameters without any constraints, except for a rigid bond restraint for the disordered carbon atoms C21, C24, C18 which is appropriate as indicated by the equal values obtained for 'rms deviation' and 'rms sigma' upon refinement. The data of the crystal structure are available quoting CCDC 795562**.**

Crystallographic data for 3: $C_{28}H_{40}Cl_2F_{10}N_4P_2Pd$, M_r=861.88, monoclinic, space group I 2/a, a=24.1455(10) Å, b=9.0003(4) Å, c=48.655(2) Å, β=92.825(2)°, V=10560.8(8) Å^3, Z=12, d_{calc}=1.626 g·cm^{-3}, $\mu(Mo_{K\alpha})$ =0.850 mm^{-1}, T=100K, F(000)=5232, 113786 reflections measured, 15338 unique (R_{int}=0.0403), data/parameters/restraints=15338/737/36, R1=0.0344 ($I > 2\sigma(I)$), wR2=0.0935 (all), GOF=1.034. The asymmetric unit contains two independent molecules. The non-hydrogen atoms of the molecule I containing Pd1 were refined with anisotropic displacement parameters without any constraints. The other molecule (II, containing Pd5) lying near an inversion centre is disordered over two orientations and was refined with site occupation factors of 0.5. Some additional constraints were applied for the second molecule: The C atoms of the phenyl rings were fitted to a regular hexagon with C-C distances of 1.3845 Å, the mean value of the analogous bond lengths in molecule I. The same anisotropic displacement parameters were used for the corresponding C, N, and F atoms, respectively. Furthermore in the disordered molecule II the various P-N, C-F, N-C, and C-C

(of the ethyl groups) distances were restrained to be equal in length for each bond type (DFIX of SHELXL-97) but these bond lengths were enabled to refine. The data of the crystal structure are available quoting CCDC 771838.

Crystallographic data for 4a: $C_{28}H_{40}CuF_{10}IN_4P_2$, M_r=875.03, monoclinic, space group P 2_1/c, a=16.8291(7) Å, b=11.6826(5) Å, c=18.7734(7) Å, β=104.779(2), d_{calc}=1.629 g·cm^{-3}, μ($Mo_{K\alpha}$)=1.644 mm^{-1}, F(000)=1752, 105420 reflections measured, 10398 unique (R_{int} = 0.0350), data/parameters=10398/480, R1=0.0208 (I > 2σ(I)), wR2=0.0530 (all), GOF=1.038. One diethylamino group is disordered; the site occupation factors refined to 0.628(3) and 0.372(3), respectively, for the two orientations. The data of the crystal structure are available quoting CCDC 771839.

Crystallographic data for 4b: $C_{28}H_{40}CuF_{10}IN_4P_2$, M_r=875.02, monoclinic, space group I a, Z=4, a=18.8155(11) Å, b=10.0910(4) Å, c=20.5851(8) Å, β=115.0890(10)°, V=3539.7(3) Å^3, d_{calc}=1.642 g·cm^{-3}, μ (($Mo_{K\alpha}$)=1.658 mm^{-1}, T=100 K, F(000)= 1752, 31479 reflections measured, 13206 unique (R_{int}=0.0199), data/parameters/ restraints=13206/520/16, R1=0.0162 (I > 2σ(I)), wR2=0.0385 (all), GOF=1.007. Four of the eight ethyl groups are disordered, and refined with individual site occupation factors. The data of the crystal structure are available quoting CCDC 771840.

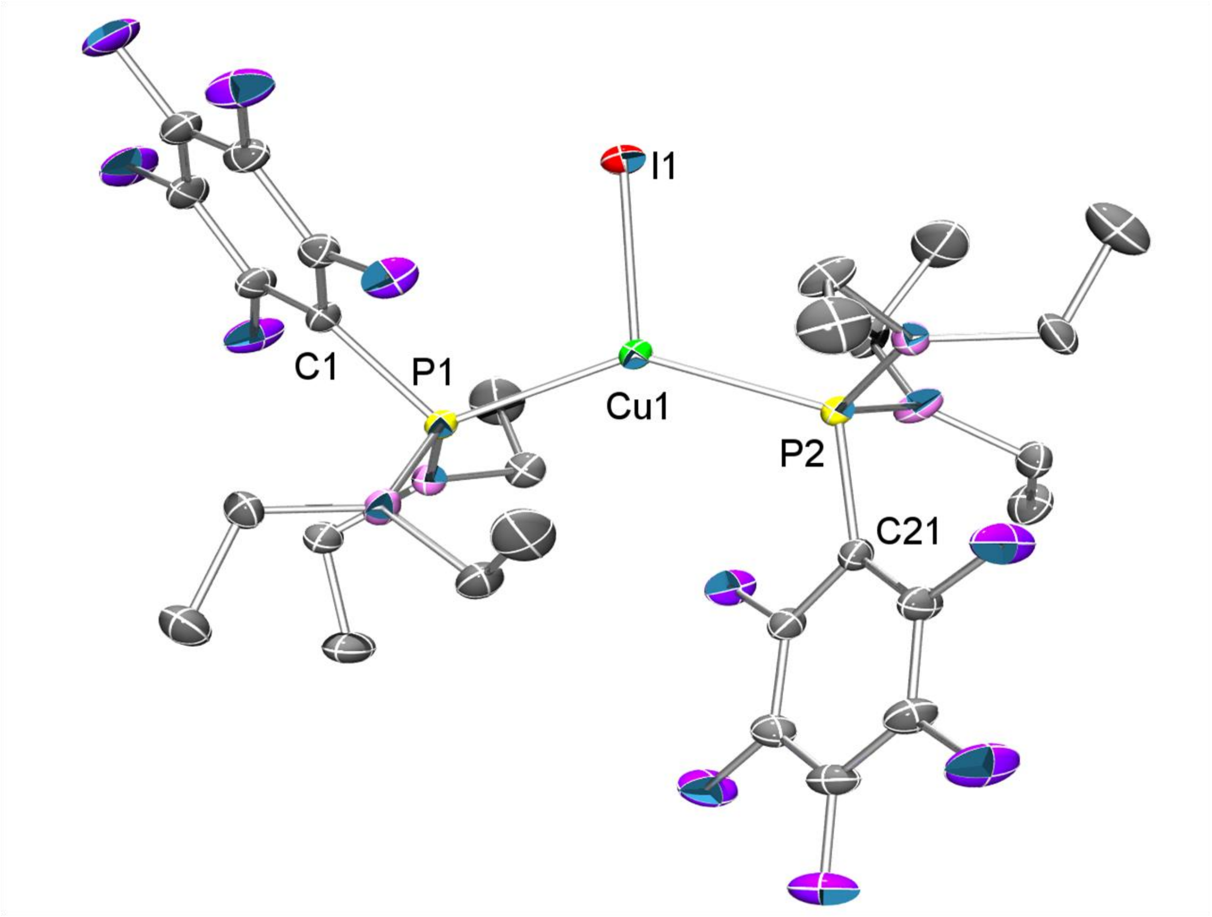

Crystal structure of **4b**

Crystallographic data for 4c: 2 ($C_{28}H_{40}CuF_{10}IN_4P_2$)*$C_4H_8O$, M_r=1822.14, monoclinic space group P 2/c, Z=4, a=25.4888(9) Å, b=18.3825(6) Å, c=16.3908(6) Å, β=100.3930(10)°, V=7553.9(5) Å^3, d_{calc}=1.602 g·cm^{-3}, μ($Mo_{K\alpha}$)=1.558 mm^{-1}, T=100 K, F(000)=3664, 135615 reflections measured, 21718 unique (R_{int}=0.0279), data/parameters/restraints = 21718/1122/368, R1=0.0357 (I > 2σ(I)), wR2 = 0.0753 (all), GOF=1.273. Two of the four pentafluorophenyl rings, one ethyl group of a diethylamino group, and one of the two tetrahydrofuran molecules are disordered over two orientations each and were refined with site occupation factors of 0.5. The equivalent bonds in the disordered pentafluorophenyl rings and in the disordered ethyl group are restrained to have the same lengths. The equivalent bonds in the disordered tetrahydrofuran molecule were restrained to the bond lengths in the ordered molecule. The data of the crystal structure are available quoting CCDC 771841.

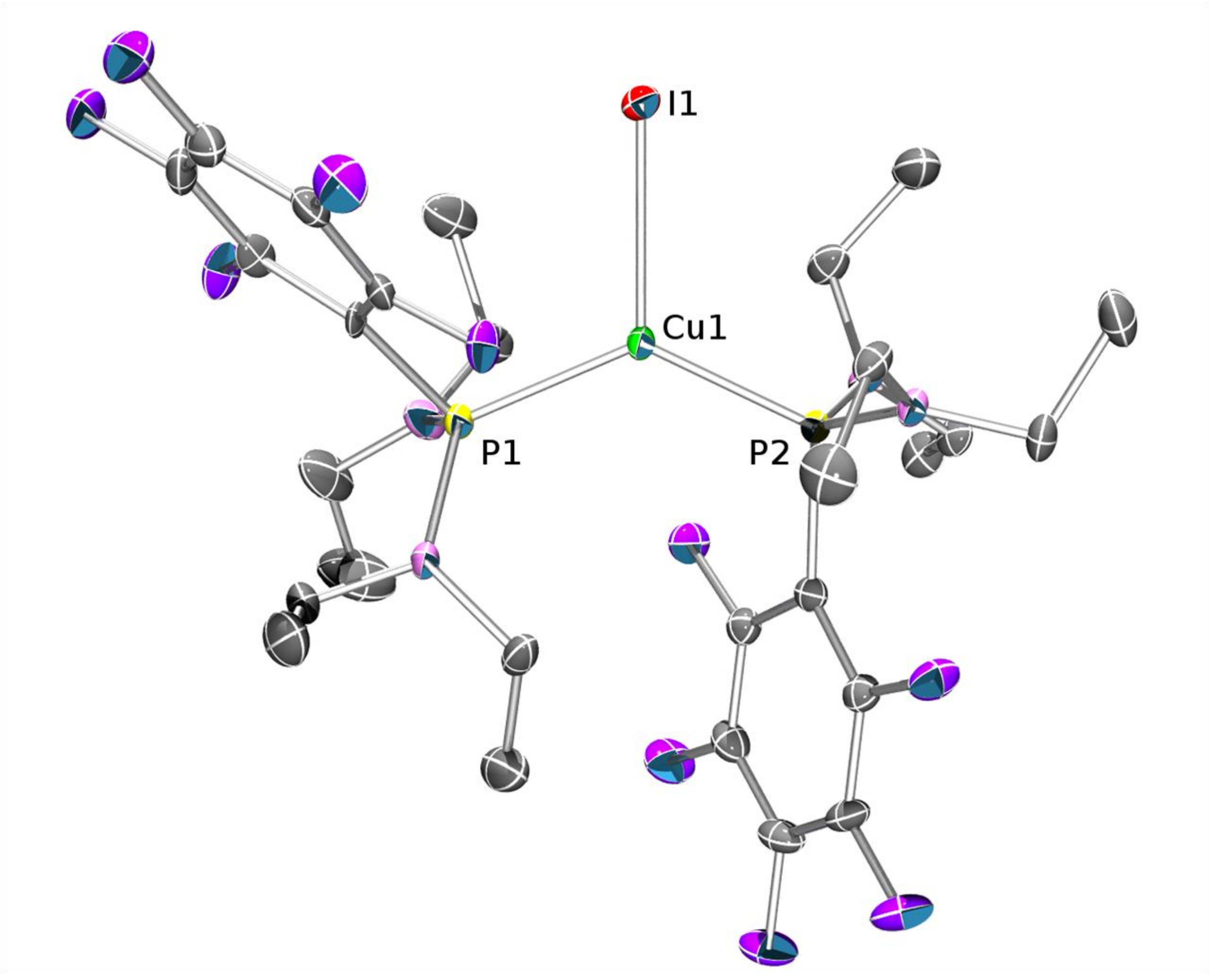

Crystal structure of **4c**

Catalytic data

Suzuki-Miyaura type cross coupling (Scheme 4 in manuscript)

Reaction conditions and yields are provided in Table 16.

Entry	Time [h]	Conv. [%][a]		
		$PdCl_2$	$Pd(PPh_3)_2Cl_2$	**2**
1	6	< 1	12	23
2	24	12	31	51
3	30	18	35	61
4	48	34	43	74
5	60	40	51	76
6	72	52	59	76
7	96	55	69	77
8	120	58	73	79
9	168	66	79	90

Table **10** General reaction conditions: 1.0 mmol 4-iodobenzonitrile, 1.2 equiv. boronic acid, 1.5 equiv. potassium carbonate, 1.0 mol% catalyst in 2.0 mL of toluene room temperature; [a] GC-FID conversions are reported.

References

1. A. D. Becke, *The Journal of Chemical Physics*, 1993, **98**, 5648-5652.
2. C. Lee, W. Yang and R. G. Parr, *Phys. Rev. B: Condens. Matter*, 1988, **37**, 785-789.
3. P. J. Hay and W. R. Wadt, *J. Chem. Phys.*, 1985, **82**, 270-283.
4. P. J. Hay and W. R. Wadt, *J. Chem. Phys.*, 1985, **82**, 299-310.
5. M. J. Frisch, G. W. Trucks, H. B. Schlegel, G. E. Scuseria, M. A. Robb, J. R. Cheeseman, J. A. Montgomery, Jr, T. Vreven, K. N. Kudin, J. C. Burant, J. M. Millam, S. S. Iyengar, J. Tomasi, V. Barone, B. Mennucci, M. Cossi, G. Scalmani, N. Rega, G. A. Petersson, H. Nakatsuji, M. Hada, M. Ehara, K. Toyota, R. Fukuda, J. Hasegawa, M. Ishida, T. Nakajima, Y. Honda, O. Kitao, H. Nakai, M. Klene, X. Li, J. E. Knox, H. P. Hratchian, J. B. Cross, C. Adamo, J. Jaramillo, R. Gomperts, R. E. Stratmann, O. Yazyev, A. J. Austin, R. Cammi, C. Pomelli, J. W. Ochterski, P. Y. Ayala, K. Morokuma, G. A. Voth, P. Salvador, J. J. Dannenberg, V. G. Zakrzewski, S. Dapprich, A. D. Daniels, M. C. Strain, O. Farkas, D. K. Malick, A. D. Rabuck, K. Raghavachari, J. B. Foresman, J. V. Ortiz, Q. Cui, A. G. Baboul, S. Clifford, J. Cioslowski, B. B. Stefanov, G. Liu, A. Liashenko, P. Piskorz, I. Komaromi, R. L. Martin, D. J. Fox, T. Keith, M. A. Al-Laham, C. Y. Peng, A. Nanayakkara, M. Challacombe, P. M. W. Gill, B. Johnson, W. Chen, M. W. Wong, C. Gonzalez and J. A. Pople, in *Gaussian, Inc., Pittsburgh PA*, 2003.
